# Contextual recommendation modeling in eCoaching with machine learning, X-AI, and semantic ontology

**DOI:** 10.3389/fdgth.2026.1811976

**Published:** 2026-07-15

**Authors:** Ayan Chatterjee, Nurilla Avazov

**Affiliations:** 1 Stiftelsen NILU, Department of Digital Technology, Kjeller, Norway; 2 Inland School of Business and Social Sciences, University of Inland Norway, Lillehammer, Norway

**Keywords:** classification, context-aware recommendations, eCoaching, explainable AI, semantic ontology mapping

## Abstract

Physical activities can be divided into indoor and outdoor activities. While outdoor activities offer enjoyable fitness opportunities, they are often limited by weather conditions. Unfavorable weather conditions such as cold, rain, fog, or snow can significantly re­duce physical activity levels, posing risks such as heat stress, dehydration, or cold-related injuries. To address these challenges, we have developed the concept of an automated eCoaching system that provides personalized activity recommendations based on real-time weather data. Our system uses an algorithm to annotate, process, and classify the collected data, generating tailored suggestions for indoor or outdoor exercise. This information is semantically represented using an Ontology framework. We have conducted a comprehensive study by collecting weather data for 18 months from thirteen cities in southern Norway. Furthermore, we have developed rules to determine the appropriate activity types corresponding to different weather conditions. The classification performance of the system has been rigorously evaluated using metrics such as accuracy, precision, recall, F1 score, and Matthews correlation coefficient (MCC). Remarkably, the decision tree classifier achieved an accuracy of 99.1%. To increase interpretability, we used local model-independent interpretable explanations (LIME) to explain individual predictions. The consistency of the Ontology model has been verified using inference, providing a reliable semantic representation and efficient rule-based recommendation modeling. In addition, we have developed various test cases of the system to evaluate eCoaching recommendations under different weather scenarios. This approach provides users with accurate and contextually relevant guidance, promoting continuous physical activity regardless of external weather conditions.

## 1 Introduction

### 1.1 Background

Between 60% and 85% of the population in both developed and developing countries lead a sedentary lifestyle (1). A sedentary lifestyle is linked to adverse health outcomes, including higher risks of obesity, diabetes, hypertension, and cardiovascular diseases (2–6). Sedentary individuals face a 20% to 30% higher risk of death than active individuals (7). Regular physical exercise mitigates these risks, with the WHO recommending 150–300 min of moderate or 75–150 min of vigorous aerobic activity weekly (8). Environmental factors, particularly severe weather, pose barriers to consistent physical activity (9, 10). Seasonal variations, including extreme temperatures, rain, and snow, discourage outdoor activities (9). Providing indoor options during adverse weather can support year-round activity (10). Studies highlight how weather influences activity, with decreased participation during winter (11, 12). Meteorological factors, such as temperature, rain, and wind, are significant barriers to physical activity (10, 11). Seasonal variations impact activity levels, underscoring the need to mitigate weather-related challenges (12, 13). This study analyzes how weather changes impact indoor and outdoor activities seasonally. Weather data from 13 cities in Southern Norway were collected over 1.5 years using the Open-Weather interface (14). Southern Norway experiences four distinct seasons, with average annual temperatures of 7.5 °C and rainfall of 1,260 mm (15). In Norway, approximately 70% of adult men and around 60% of adult women are classified as overweight, with obesity-related mortality contributing to approximately 8% of total deaths in 2016 (16, 17). These figures primarily reflect the adult population rather than the entire national demographic. Poor weather exacerbates inactivity, especially among elderly individuals (18). An eCoaching system can provide personalized, weather-aware recommendations to promote physical activity. Such systems can track weather, set goals, and generate contextual recommendations, encouraging indoor or outdoor activity based on conditions (19). eCoaching platforms combine user-friendly interfaces with clinical guidelines and reasoning capabilities to support sustained activity (20). Automatic interventions offer an effective alternative to traditional coaching by leveraging data-driven insights for personalized recommendations.

### 1.2 Research gap

Although many eCoaching systems support behavior change, most provide generic guidance and rely mainly on user activity history, with limited use of contextual factors. Existing studies on weather and physical activity largely describe correlations, but do not translate real world weather dynamics into personalized indoor or outdoor exercise recommendations. In addition, current approaches rarely combine machine learning-based activity type classification (e.g., predicting whether indoor or outdoor activities are suitable under given weather conditions) with explicit semantic knowledge models. As a result, the decision logic remains opaque, limiting interpretability, structured reasoning, and the reuse of recommendations across varying contextual scenarios. Our study addresses these gaps by integrating long-term multi-city weather data (e.g., temperature, humidity, precipitation, wind speed, and air quality) with personal preferences (e.g., preferred activity type, time of day, notification mode) and physiological signals (e.g., daily step count, moderate-to-vigorous physical activity duration, sedentary time) to generate context-aware recommendations. For example, if forecasted heavy rain coincides with low weekly activity progress, the system may recommend light indoor aerobic exercise; conversely, mild temperature and clear sky conditions combined with a sedentary activity profile may trigger a recommendation for outdoor walking or cycling. We annotate and classify weather conditions using machine learning, explain predictions with LIME, and represent the knowledge in an ontology to enable consistent reasoning and rule driven recommendation generation. This hybrid design improves personalization, interpretability, and reliability of activity guidance under changing seasonal and meteorological conditions. Throughout this paper, the term *eCoach* refers to the proposed intelligent recommendation system, whereas *eCoaching* denotes the broader conceptual framework of digital behavioral intervention.

### 1.3 Motivation, research questions, and aim of the study

The motivation behind this study is the increasing prevalence of chronic lifestyle-related diseases such as obesity and overweight. Personalized eCoaching has the potential to help individuals make lasting changes in their health behaviors. By improving the personalization and contextual relevance of advice, we aim to increase user engagement and the overall effectiveness of eCoaching interventions. Current eCoaching systems often lack the ability to fully integrate and interpret a variety of data sources, such as real-time activity levels, environmental factors, and personal preferences, to create truly personalized recommendations. This limitation can result in generalized recommendations that fail to effectively motivate or engage users. To address the identified limitations in current eCoaching systems, this study investigates the following research questions:

- RQ1: How can contextual weather data, physiological signals, and personal preferences be integrated to improve personalized recommendation generation in eCoaching systems?
- RQ2: How can explainable machine learning and semantic ontology improve transparency, reasoning capability, and contextual recommendation generation?
- RQ3: How can ontology-driven reasoning and SPARQL querying support scalable and logically consistent recommenda­tion generation under dynamic environmental conditions?

In this study, we have developed an advanced eCoaching system that generates personalized recommendations for indoor or outdoor activities by integrating multimodal data, such as weather conditions, physiological signals, and user preferences with predictive modeling. Furthermore, we have developed an algorithm to annotate, process, and classify weather data to generate adaptive, personalized, targeted, and rule-based recommendations. Furthermore, to improve knowledge representation, we have created a domain ontology for semantic annotation to facilitate data integration, reasoning, and querying. We have used an Explainable AI (X-AI) method, such as LIME to explain the classification results and used SPARQL Protocol and RDF Query Language (SPARQL) queries to generate rule-combined hybrid recommendations against the knowledge base. SPARQL is a semantic query language used to retrieve and manipulate structured data stored in Resource Description Framework (RDF) knowledge graphs. This approach aims to provide adaptive, personalized, goal- and rule-combined hybrid recommendations that improve user compliance and health outcomes. By addressing the identified research gaps, this study contributes to the field of personalized health technology and provides insights for developing more effective eCoaching systems that can adapt to the unique contexts and needs of individual users. This study proposes a novel eCoaching system that generates personalized activity recommendations by analyzing contextual weather data. Traditional ontologies such as RDF and W3C Web Ontology Language (OWL) (21–25) lack native support for complex production rules commonly found in rule-based systems. To address this issue, we adopt a hybrid approach that combines ontologies with rule-based knowledge bases to improve structured knowledge representation, semantic reasoning, and user modeling. This integration improves the recommendation process and the overall user experience. Our literature review shows that existing eCoaching systems do not provide personalized recommendations based on contextual weather data, which highlights the novelty of our contribution. Our present study has extended our previous studies (21–25). The novelty of this study and how this study differs from our previous studies have been elaborated with a qualitative comparison and listed in Table 1.

**Table 1:** A comparison between our previous studies and this extended study.

Study	Study focus	Dataset used	Recommendation type	Method focus
A Chatterjee et al. (21)	Conceptualized the idea of weekly activity fore-casting with statistical models and a rule-base for personalized rule-based recommendation gener­ation in activity eCoaching.	PMData	Personalized	ARIMA, SARIMA, Kalman Filter, Rule-database
A Chatterjee et al. (22)	Conceptualized the idea of weekly activity fore-casting and a rule-base for personalized recom­mendation generation with Ontology reasoning and querying in activity eCoaching.	PMData	Personalized	LSTM, Ontology
A Chatterjee et al. (23)	Semantic ontology model to annotate the machine learning (ML)-classification outcomes and personal preferences to conceptualize personal­ized recommendation generation with a hybrid approach in activity eCoaching with a focus on transfer learning approach to improve ML model training and its performance, and an incremen­tal learning approach to handle daily growing data and fit them into the ML models (Support Vector, Naive Bayes, Decision Tree, K-Nearest Neighbour, Random Forest).	Zenodo Fitbit and MOX2-5	Personalized	Standard ML classification models, On­tology
A Chatterjee et al. (24)	Design and development of an extended ontology model for semantic representation of per­sonal and personalized activity data, and algo­rithm development to include time-series fore­casting, time-series physical activity level clas­sification, and statistical metrics in the ontology model for hybrid recommendation generation with person-based heuristic configuration and the verification of the algorithm against different datasets with existing and derived metrics.	PMData and MOX2-5	Personalized	Deep learning models, Ontology, Prob­abilistic Interval Pre­diction, Statistical Metrics
A Chatterjee et al. (25)	Design and development of an extended ontology model for semantic representation of per­sonal and personalized activity data, and algo­rithm development to include time-series fore­casting, time-series physical activity level classi­fication, data balancing, and traditional metrics in the ontology model for hybrid recommenda­tion generation with person-based heuristic con­figuration and the verification of the algorithm against different datasets with existing metrics.	PMData and MOX2-5	Personalized	Auto-regression, Machine learning models, ADASYN, Ontology, Probabilistic Interval Prediction, tradi­tional Metrics
Proposed work	Design and development of an extended ontology model for semantic representation of con­textual and personal preference data, algorithm development to include activity type classifica­tion, ontology model for hybrid recommenda­tion generation with person-based heuristic con­figuration, and the verification of the algorithm against different contextual datasets with exist­ing metrics.	Contextual external weather dataset	Personalized	Machine learning models, Ontol­ogy, LIME, and traditional Metrics

**Figure 1:**
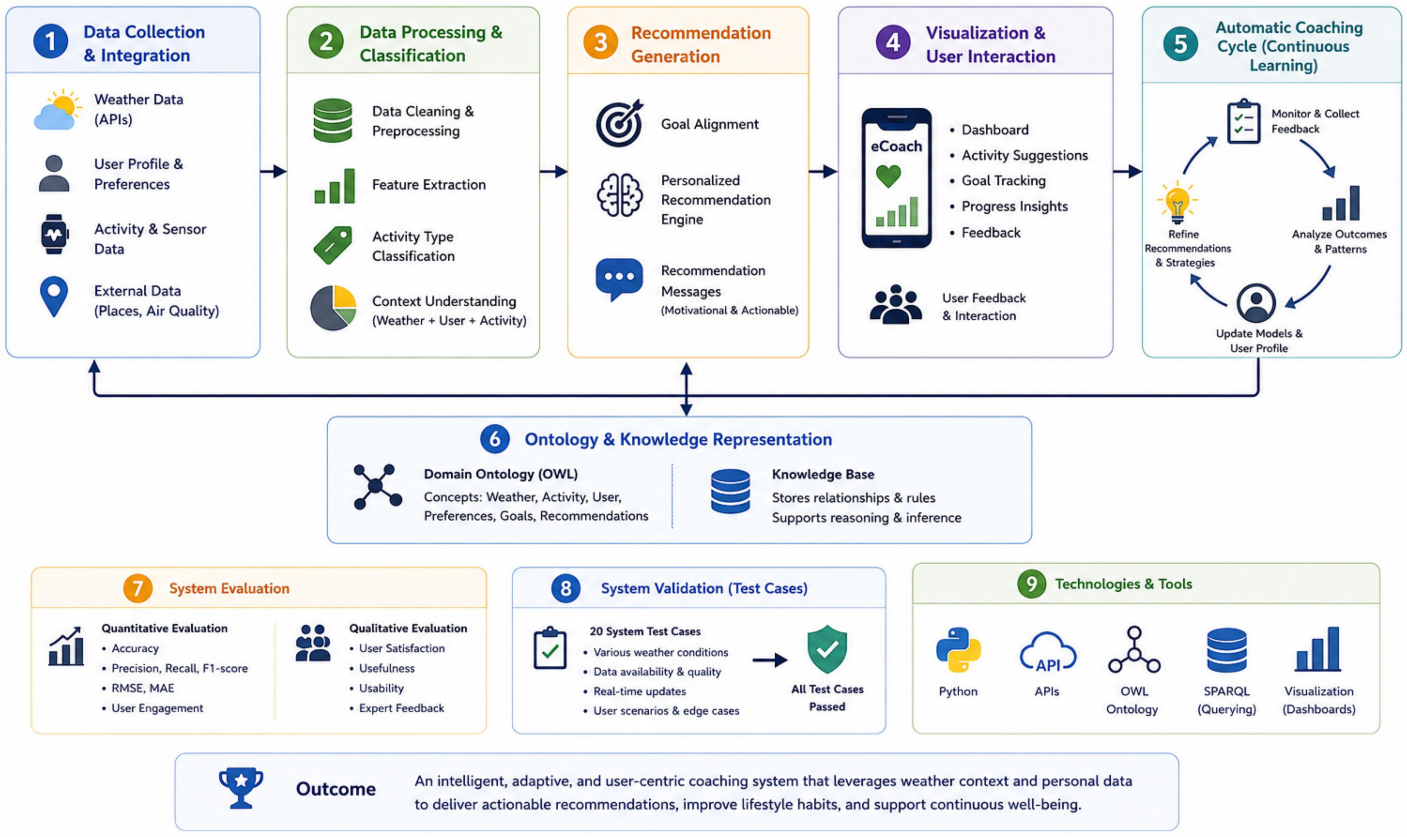
Overall framework of the proposed context-aware eCoach system illustrating data collection, activity-type classification, personalized recommendation generation, ontology-based reasoning, user interaction, and continuous coaching adaptation.

Figure 1 presents the overall architecture and workflow of the proposed eCoach framework. The system integrates contextual weather data, machine learning-based activity-type classification, ontology-driven semantic reasoning, personalized recommendation generation, and continuous feedback adaptation within an automatic coaching cycle.

### 1.4 Study distribution

To ensure the effectiveness of generating physical activity recommendations in the eCoaching system, we incorporated various test cases. The study is structured as follows−Section 2 details the eCoach system and OWL ontology, enhancing knowledge through semantic annotation, reasoning, and rule-based decision-making. Section 3 presents the methodology, including ethical approval, data collection, feature engineering, data labeling, machine learning classifier selection, classification explanation models, evaluation strategies, problem formulation, and the proposed recommendation algorithm to address the research question. Section 4 presents the feature selection, classification results with explanations, and validates the ontology model for personalized rule-based recommendations. Section 5 explores the validity of the findings in eCoaching for context-aware recommendation planning. Finally, Section 6 summarizes the study's findings. This structure provides a comprehensive understanding of our approach to personalized activity recommendations in eCoaching based on contextual weather data.

## 2 Proposed system design

This section elaborates on data flow in our eCoaching prototype system, the high-level structure of an Ontology, and an algorithm for context-based recommendation generation.

### 2.1 Ecoaching system design

A health eCoaching system (19) is an electronic platform designed to support individuals in changing and maintaining a healthy lifestyle through personalized guidance, counseling, and education. The system can integrate various technologies such as mobile apps, wearable devices, and online portals to help individuals track their health behaviors, set goals, and receive feedback and support. A health eCoaching system can be customized according to each user's specific needs and preferences (his health goals, current health status, and preferred communication channels) (20). The system can also use various data sources (user-generated data, sensor data, and health records) to provide personalized recommendations and feedback. The primary goal of a health eCoaching system is to empower individuals to take control of their health by providing them with the tools and resources they need to make informed decisions about their lifestyle behaviors. The system can also help healthcare providers deliver more personalized and effective care by using technology to monitor and support patients' health behaviors and progress. A health eCoaching system can employ various decision-making approaches to support individuals in achieving their wellness goals. The common decision-making approaches are the Goal-oriented approach, Action-oriented approach, Feedback-oriented approach, Self-reflection approach, Motivational approach, Evidence-based approach, Behavioral approach, Collaborative approach, and Strengths-based approach. The approaches are further elaborated in Supplementary Material S1. These different decision-making methods can be used in combination to provide a personalized and effective approach to recommendation generation for eCoaching. The specific method used depends on individual goals, preferences, and needs. In this study, we have combined goal-oriented, action-oriented, and evidence-based approaches.

**Figure 2:**
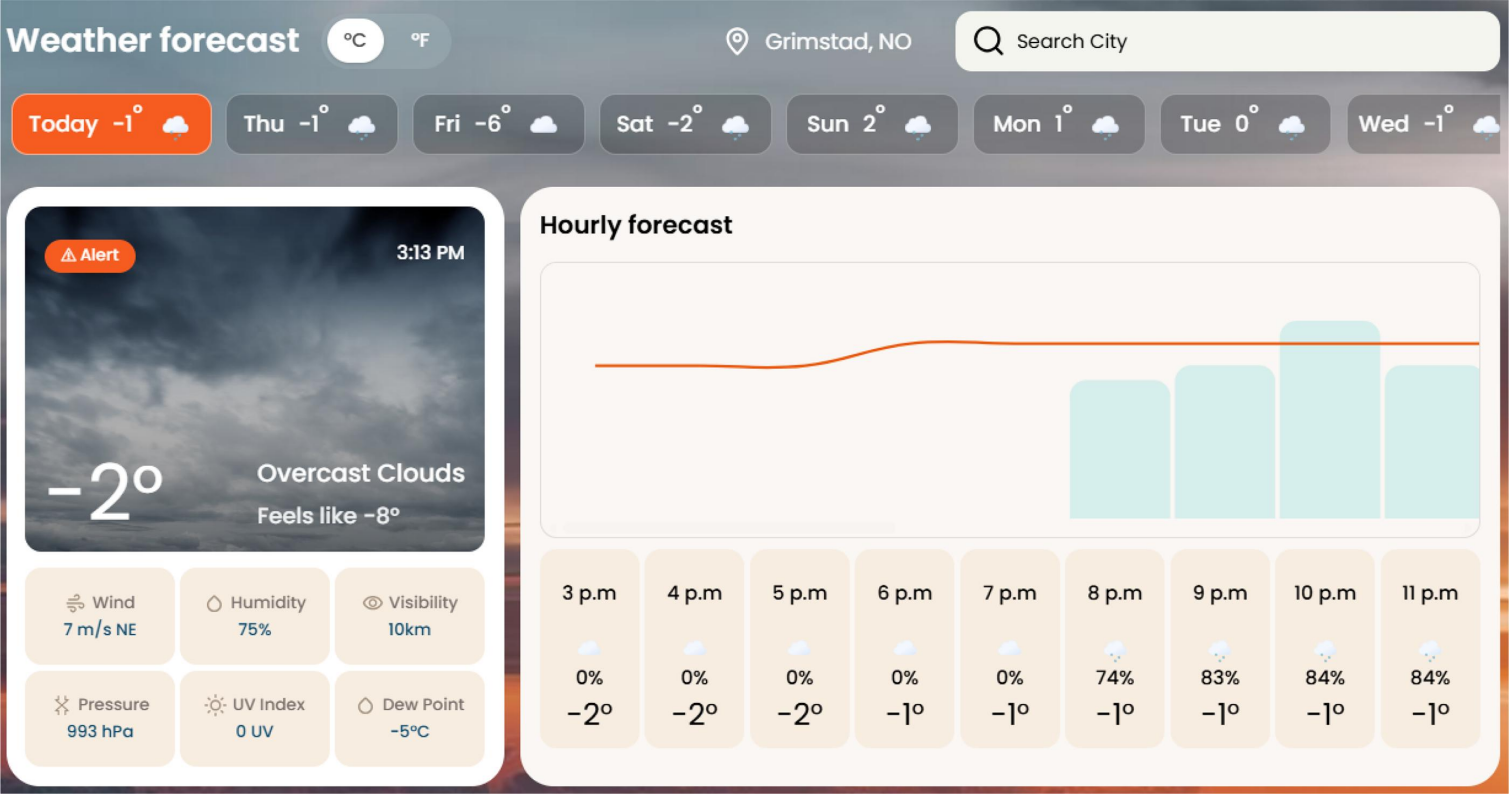
Enhanced high-level architecture of the proposed eCoach system. The framework integrates contextual weather data (sun/cloud), user activity signals (walking, sitting, standing; indoor/outdoor status), semantic processing, and recommendation generation. The final module delivers adaptive graphical and textual feedback (e.g., “go outside” or “perform light indoor activity”), similar to commercial fitness platforms such as TrainingPeaks.

**Figure 3:**
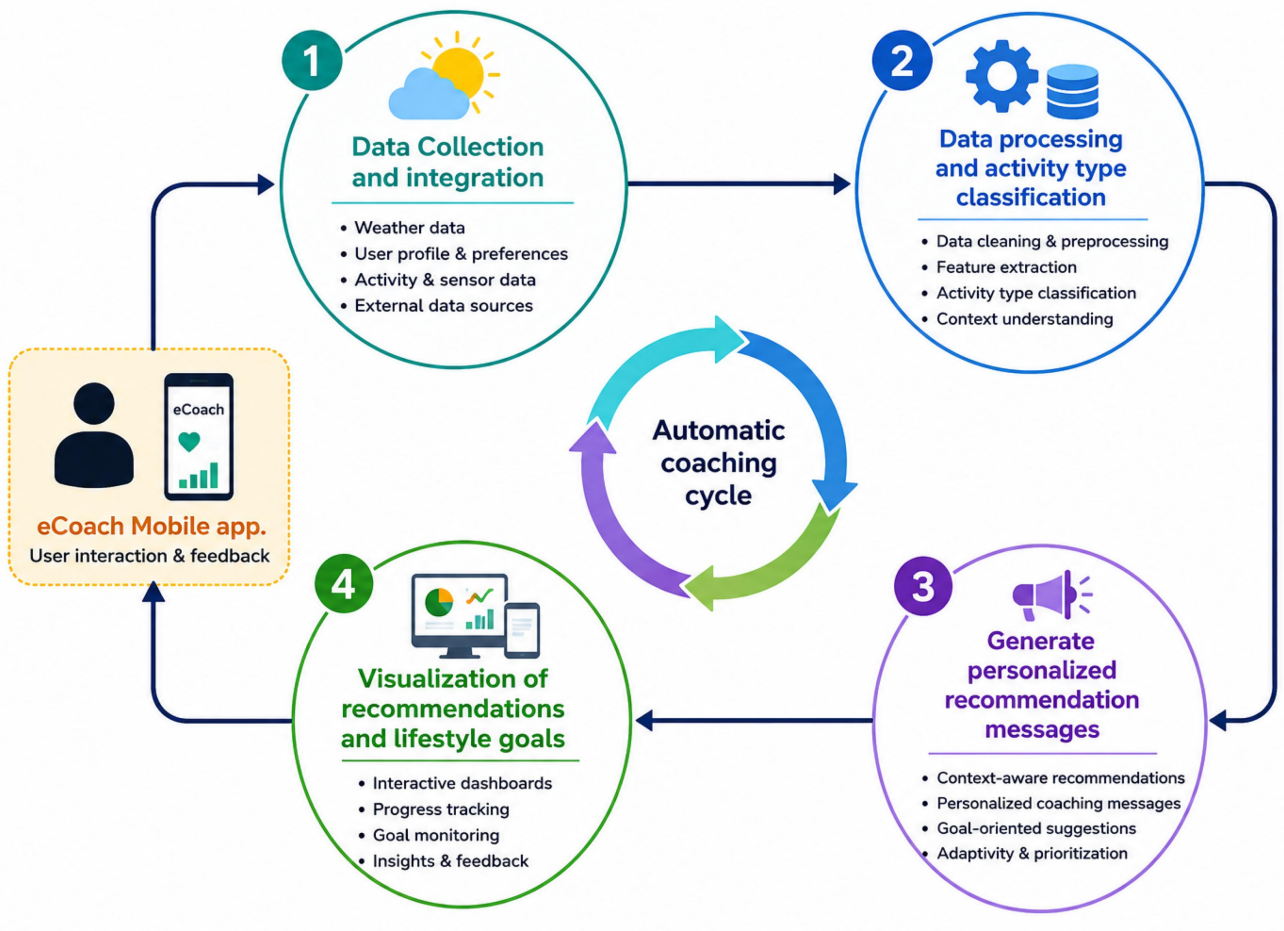
Weather prediction with openWeatherMap for grimstad, Norway.

Figure 2 has been conceptually extended to illustrate multi-modal inputs including (i) environmental signals (e.g., sun, rain, cloud), (ii) user activity posture and motion states (e.g., walk, sit, stand; indoor/outdoor detection), and (iii) semantic reasoning modules. The data can be the following three types−daily activity, contextual weather, and personal preferences. Daily activity data keeps track of daily physical activities performed by the participants. Contextual weather data accurately forecast external temperature, wind, precipitation, humidity, snow, solar, and climate summaries. Participants' preference data informs about the mode and time of recommendation delivery. The data collection, activity type classification, and recommendation generation are continuous processes according to the eCoach feedback cycle. The visualization layer represents adaptive behavioral nudges, such as recommending outdoor walking during mild sunny conditions or light indoor stretching during heavy precipitation. In this study, we consider only contextual external weather data to classify potential activity types (indoor and outdoor) for recommendation generation. The usage of the weather forecast data for activity recommendations may allow the recommendation system to be more context-aware, adapting to users' preferences and dynamic environmental conditions, leading to increased user satisfaction and engagement. We use the freely available OpenWeatherMap APIs for external weather data collection (14) (see Figure 3). OpenWeatherMap is an online service provided by OpenWeather Ltd that provides global weather data through an API, including current weather data, forecasts, and historical weather data for any geographic location (14). The company provides up-to-date precipitation forecasts for any location. The OpenWeatherMap has been our popular choice for accessing weather data due to its wide coverage, detailed data (e.g., current weather conditions, hourly forecasts, and daily forecasts), freely available APIs (e.g., a free plan that allows developers to access up to 60 calls per minute), ease of use, extensive documentation, easy file formats, and community support.

In this theoretical and experimental study, we conceptualize the design of the pop-up recommendation delivery in an Android smartphone view using Figma following the *Google Material Design*. Figma (26) is a collaborative web application for interface design with additional offline capabilities enabled by desktop applications for macOS and Windows. Figma and Google Material Design offer some useful benefits for UX designers. Figma's real-time collaboration, prototyping, vector-based, and cloud-based approach can increase efficiency and productivity, while Google Material Design's guidelines for consistency, accessibility, flexibility, cross-platform, and open-source nature can help create a more seamless user experience. Figma offers a range of third-party integration to streamline their workflow and improve collaboration.

We propose an algorithm for contextual recommendation planning (e.g., grouping, generation, and delivery) in our eCoach system by combining contextual weather data, machine learning outcomes for activity type, personal preference data (e.g., activity goal-setting, response type, and the nature of interaction), and semantic Ontology. We consider activity goal-setting as “moderate weekly active”, response type as “pop-up notification”, and the nature of the interaction as “graphical illustration and textual messaging”. We assume recommended delivery at every hour (customizable) between 9.00 am. to 8.00 pm. Participants can view and update their preference information at any time. We developed a pipeline to process growing weather data in real time through an incremental approach, automating the process. The design follows a modular microservice architecture. The recommendation generation module executes the scheduler intermittently to query and process individual campaign prediction results from the knowledge base using the SPARQL query engine. The exposed eCoach interfaces are protected by multifactor authentication and authorization (OAuth 2.0) rules (27, 28).

### 2.2 Ontology design

Semantic ontology refers to the formal representation of relations and concepts in a specific knowledge domain. It is a structured method of organizing information and knowledge by defining and describing terms and concepts and establishing their interrelationships. In an ontology, terms, and concepts are represented by nodes, and the relationships between them are represented by edges or connections between nodes. Ontologies can be hierarchical, where concepts are organized in a tree-like structure, or they can be networked, where concepts are linked in a more complex network-like structure. Ontologies can be represented mathematically using set theory and graph theory. In this representation, concepts in an ontology are depicted as a set, while the relationships between concepts are represented by directed edges connecting the corresponding sets.

Let O be the ontology, C be the concept set in O, and R be the relation set between concepts in O. We can then mathematically represent O as a directed graph G = (V, E), where V = C is a vertex or node and E = R is a set of directed edges or connections between nodes. Formally, we can define ontology O as: O = (C, R); Where C is the concept set in the ontology, and R is the relationship set between concepts in the ontology (29, 30). Each concept in an ontology can be represented as a set of instances or individuals belonging to that concept. For example, if an ontology contains the concept “city”, the set of instances belonging to that concept might contain “Grimstad”, “Arandal”, “Kristiansand”, etc. Similarly, each relation between concepts can be represented as a directed edge between corresponding instance sets. For example, if the ontology contains the relation “is a child of”, then there exists a directed edge from the set of instances belonging to the concept “child” to the set of instances belonging to the concept “parent”. Overall, mathematical representations of ontologies provide a formal way to reason about concepts and relationships in ontologies and to develop algorithms for tasks such as classification, retrieval, and reasoning. In ontologies, A-box and T-box refer to two different types of knowledge representations for defining and describing concepts in a domain. T-Box (Term Box) represents the connotative knowledge of ontology and contains a set of axioms that define concepts and relationships within the domain. T-box is used to represent the schema or structure of an ontology and is usually expressed in a formal language such as OWL (Web Ontology Language) (29, 30). On the other hand, A-Box (Assertion Box) represents the extended knowledge of ontology, containing a series of assertions that specify instances of the concepts and relations defined in the T-Box (29, 30). A-box is used to represent data or facts of an instantiated ontology, usually expressed in a triple format (subject, predicate, object), such as RDF (29, 30). Thus, the T-box defines the structure and concepts of the ontology, while the A-box defines the instances of these concepts in the ontology (29, 30). The T-box provides the schema for the ontology, while the A-box provides the data or facts that instantiate the schema (29, 30). In Ontology, a knowledge base refers to a collection of information used to represent a domain of knowledge. A knowledge base contains the T-box and A-box components of an ontology, which together define concepts, relations, and instances in the domain. Ontology development is a complex process involving multiple steps and methods. The following are the general steps for ontology development used in this study −

- Domain identification to define the scope of Ontology (O) and model the concepts and relationships.
- Knowledge gathering on the domain, including relevant literature, expert opinions, and existing ontologies. This information is used to identify concepts and relationships that need to be modeled in the ontology.
- Defining Ontology structure with classes, properties, and relationships that will be used to represent the concepts and their interrelationships.
- Ontology development with Ontology editors or other software tools. This involves creating the classes, attributes, and relationships defined in the previous step and adding instances to the ontology to illustrate the concepts.
- Checking structural consistency of the Ontology with reasoners.
- Validation of an ontology to ensure that it accurately represents the domain and can be used for its intended purpose. This may involve using ontologies to perform tasks such as classification, retrieval, or inference.
- Refine and update the Ontology with new knowledge. It is important to maintain the Ontology and ensure it remains current and useful.

**Figure 4:**
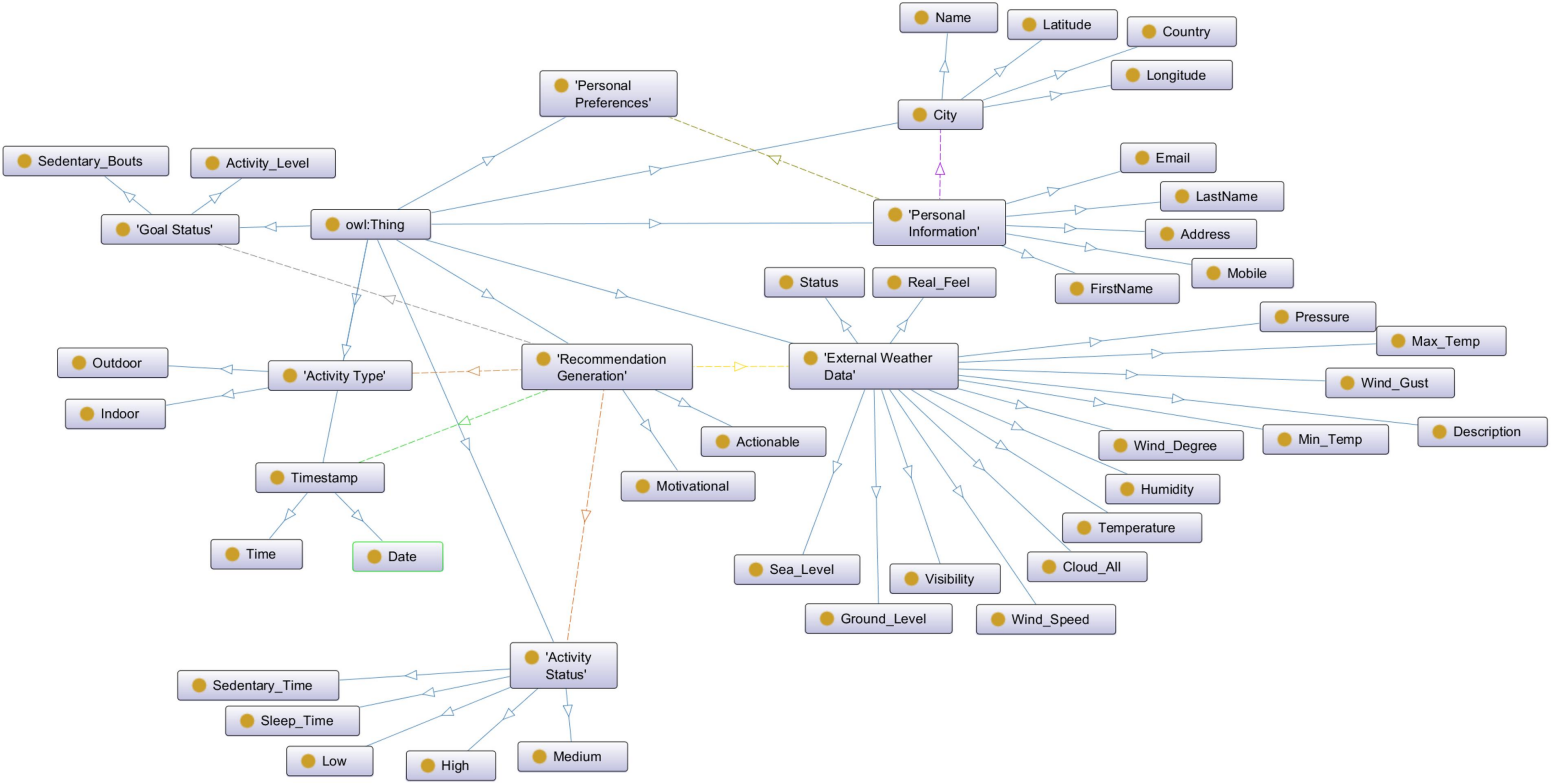
The high-level graphical structure of the designed ontology developed in protege.

Therefore, ontology development is an iterative process that involves refining and improving an ontology over time based on feedback from domain experts and users. The above algorithms provide a general framework for ontology development; however, specific steps and methods may vary depending on the domain and ontology requirements. We designed and developed an OWL Ontology model in the Protégé (v. 5.x) open-source software to represent collected data, classification outcomes, and recommendations in a meaningful way so that knowledge can be reasoned, and necessary information can be queried with SPARQL query engine. Web Ontology Language (OWL) is a language based on computer logic, so knowledge expressed in OWL can be exploited by computer programs, e.g., to verify the consistency of such knowledge or to make tacit knowledge explicit (31). We visualized the Ontology using the OWL Viz tool embedded in Protégé. In the object-oriented paradigm, owl:Thing acts as a global parent class and the arrows define a hierarchical relationship (IS-A) between the concepts (3, 32). An Ontology can be represented with the following elements: classes, objects, properties, relationships, and axioms. There are two types of properties: ObjectProperties and DataProperties. Each property has a domain scope, a restriction rule, a restriction filter, and a restriction type, such as Some (existential), Only (Universal), Min (minimum cardinality), Exact (exact cardinality), and Max (maximum cardinality). Our OWL Ontology follows the following knowledge representation phases: abstraction phase for rule mapping, abduction phase for hypothesis generation rule, deduction phase for operator-reduction rule, and induction phase for generalization rule. Our designed and developed Ontology annotates the subsequent data types for reasoning−timestamp, external weather sensor observation, personal information, and personal preference data, activity type, personalized recommendations, and participant health records (e.g., activity level) in the processed forms. The ontology metrics used in our Ontology design are−a. Metrics (Axiom (*n* = 130), Logical axiom count (*n* = 58), Declaration axiom count (*n* = 60), Class count (*n* = 46), object property count (*n* = 8), Data property count (*n* = 10) and Annotation property coun (*n* = 8)), b. Class axioms (SubClassOf (*n* = 37), c. Object property axioms [ObjectPropertyDomain (*n* = 8), ObjectPropertyRange (*n* = 8)], d. Data property axioms [DataPropertyDomain (*n* = 10), and DataPropertyRange (*n* = 10)], and Annotation axioms [AnnotationAssertion (*n* = 20)]. “n” signifies counts ≥ 0. The high-level structure of the Ontology has been depicted in Figure 4. This ontology includes classes for City, PersonalPreferences, PersonalInformation, ExternalWeatherData, ActivityType, Timestamp, GoalStatus, ActivityStatus, and RecommendationGeneration. It also includes properties that describe relationships between these classes, such as hasPersonalPreferences, hasPersonalInformation, hasExternalWeatherData, hasActivityType, hasTimestamp, hasGoalStatus, hasActivityStatus, and generatesRecommendation. This ontology model has been used to develop an eCoaching system that generates personalized activity recommendations based on a user's personal information, location, weather data, and preferences. Here, we have used operators like NOT, AND, OR, IMPLIES, EQUIV, and quantifiers (a set of logical quantifiers in a given logic) to represent complex relationships and constraints among concepts in our ontology. In this study, we use FOR ALL as a universal quantifier and EXISTS as an existential quantifier to define the scope of relationships across concepts. Quantified clauses (sets of propositional variables connected by an operator and a quantifier) and clauses (quantified clauses without a quantifier) enable the representation of intricate logical statements. Furthermore, formulas, which link clauses and quantified clauses together by logical operators, and the process model (a set of assignments to each propositional variable such that the process yields the constant TRUE when simplified) facilitate the reasoning process within the ontology. These terms and concepts play a crucial role in the presentation and processing of our ontology, as they allow for efficient reasoning and extraction of meaningful information from the knowledge base, ultimately enhancing the performance of the eCoaching system in generating personalized activity recommendations. The following terms have been used for our Ontology presentation and processing (32)−propositional variables, constants, and operators. Databases excel at efficiently storing, retrieving, and managing large volumes of structured data, especially when complex reasoning and semantic relationships are not a primary concern. Using an ontology over a traditional database offers the ability to provide a more semantic and flexible representation of knowledge. The choice between ontology and a database should be based on the specific requirements of the application and the nature of the data to be managed. In many real-world scenarios, a hybrid approach that combines the strengths of both ontology and databases proves to be the most effective solution. Here, we have approached the same with an OWL Ontology and Rule database as a knowledge base.

### 2.3 Problem formulation and recommendation generation algorithm

According to WHO guidelines, the maximization problem is to stay moderately active weekly (*Σ* ModerateActivitytime *>* 150 min). Personalized recommendation modeling based on weather data can help participants with suggestions for indoor and outdoor activities and an enumerable amount of physical activities. In this binary classification problem, sufficient daily indoor/outdoor activity scored X, and insufficient indoor/outdoor activity earned a score of Y (X *>* Y) to turn the weekly goal score target to 7 ∗ *X* (*Σ* GoalScoredaily=7 ∗ *X)*. We used preference, weather information, and activity types for recommendation generation. The customizable recommendation table (RT) for recommendation generation has been described in the Supplementary Material S2. The Algorithm 1 describes a contextual activity type recommendation process in eCoaching with a meta-heuristic approach. For verification, we used contextual weather test data to cover the following two potential cases for recommendation generation as a part of Ontology verification−*Σ* GoalScoredaily *<*7 ∗ *X* and *Σ* GoalScoredaily ≥7 ∗ *X*. We plan each recommendation to contain two types of messages−Formal (activity type to be performed based on weather information), and Informal (praising or motivational notes based on the weekly goal score achieved).

Our Ontology-based custom recommendation generation rule follows the structure *[(rule) IMPLIES (condition variable set)* → *recommendation message]*. In this regard, we include semantic rules, conditions with propositional logic, and recommendation messages against corresponding proposition variables. The semantic rules help to explain the logic behind recommendation generation through logical *AND*, *OR*, and *NOT* operations. Moreover, we added the rule for satisfiability to ensure finite and non-overlapping execution of recommendation messages. All rule execution in the Ontology structure internally follows a binary tree structure, where the non-leaf nodes contain the semantic rules to be executed (*X* | *X* → *Y)*, and the leaf nodes have the results (X or recommendation message). The edges have a decision rule (true or false). Some examples of the propositional logic used in this external weather-based physical activity type eCoach recommendation system −

- “If the temperature is above XX degrees Celsius and the humidity is high, then recommend indoor activities or water sports.” (temperature > XX AND humidity=high) => recommend indoor activities or water sports.
- “If the UV index is above YY, then recommend applying sunscreen and avoiding outdoor activities during peak sun hours.” (UV index > YY) => recommend applying sunscreen and avoiding outdoor activities during peak sun hours.
- “If the air quality is poor, then recommend indoor activities or wearing a mask during outdoor activities.” (air quality=poor) => recommend indoor activities or wearing a mask during outdoor activities.
- “If the wind speed is above zz mph, then recommend low-impact activities or activities that are less affected by wind, such as yoga.” (wind speed > zz) => recommend low-impact activities or activities less affected by wind.
- “If there is a chance of thunderstorms, then recommend avoiding outdoor activities that involve water or being exposed to open spaces.” (chance of thunderstorms) => recommend avoiding outdoor activities involving water or open spaces.
- “If the temperature is below freezing and there is snow on the ground, then recommend winter sports like skiing, snowboarding, or snowshoeing.” (temperature < 0 AND snow on the ground) => recommend winter sports.
- “If the weather conditions are extreme or dangerous, then recommend avoiding outdoor physical activity.” (chance of extreme or dangerous) => recommend avoiding outdoor activities).
- “If the air quality is good and the temperature is mild, then recommend outdoor activities like walking, running or cycling.” (Air quality=Good AND temperature=mild) => recommend outdoor activities like walking, running or cycling).
- “If weekly goal score achieved, then recommend praising notes.” (weekly goal score=achieved) => recommend praising notes).
- “If weekly goal score not achieved, then recommend motivational notes.” (weekly goal score=not achieved) => recommend motivational notes).

To improve the effectiveness of Algorithm 1, we assess its time complexity more thoroughly. This analysis facilitates the understanding of how the algorithm's efficacy increases as the input size increases. The time complexity is typically expressed using the big O notation, which describes the maximum increase in time that the algorithm can have. By studying the complexity of time, we can assess the effectiveness and potential of the algorithm, compare the performance of different methods, and identify any limitations in design. For the proposed algorithm, the time complexity is assumed to be quadratic, that is, *O*(*n*^2^). This is due to the presence of a nested loop. Here, *n* represents the number of contextual weather observations and recommendation-processing operations evaluated during each execution cycle of the recommendation framework. This quadratic time complexity is indicative of the fact that the computational time of the algorithm grows by a power of two with the input volume.

Algorithm 1 **Contextual activity type recommendation in eCoaching with the meta-heuristic approach.**

**Input:** Individual daily activity status (e.g., activity intensity) *S(t)*; Bi-hourly weather details *W(t)*;

 Recommendation message set R

 Preference set *P={Goal setting, target goal, target activity score, mode of interaction, recommendation delivery time}*;

 Duration of eCoaching *DueCo*

**Output:** Context-based recommendation message set

 *L* ⊆ *R*

1: Days ← 0

2: **while** (Days *< DueCo*) **do**

3: *W(t-1)* ← load (previous day's daily weather data)

4: pre-process *W(t-1)* and split it into set *XY={x_train_, x_test_, y_train_, y_test_}*

5: initialize list{L}=*φ*

6: *selected_Classi_ _fier_* ← predict configuration for the ML-classifier model *(C)* with set *XY*

7: *Contextual*_*reco* ← {Δ_1_*,* Δ_2_*,* Δ_3_*,* Δ_4_*,* Δ_5_*,* Δ_6_*,* Δ_7_}

 Δ_1_=Individual activity status

 Δ_2_=Individual weekly goal score

 Δ_3_=Current weather status and classification for activity type

 Δ_4_=Upcoming weather status and classification for activity type

 Δ_5_=Achieved goal score

 Δ_6_=Compare probable delta between Δ_5_ and Δ_2_

 Δ_7_=SPARQL query processing based on Δ_3_, Δ_4_, and Δ_6_

8: update list{L} based on Δ_7_ with daily and weekly recommendations

9: *Days* ← *Days*+1

#### 2.3.1 Problem formulation with constraint programming

Let *m* denote the total number of candidate activities, and *i* represent the index of an activity in the activity set *A*={*a*_1_*, a*_2_*,* *…, a_m_*}. Each *a_i_* corresponds to a specific physical activity option. The binary decision variable *p*(*a_i_*) ∈ {0*,* 1} indicates whether activity *a_i_* is selected (1) or not (0). The function *d*(*a_i_*) denotes the duration (in minutes) associated with activity *a_i_*. The weight parameter *w_i_* represents the contextual priority or benefit score assigned to activity *a_i_* based on environmental suitability and user preference.

Let *X* = {*x*_1_*, x*_2_*,* *…, x_n_*} represent the set of daily weather data attributes such as temperature, humidity, and wind speed. The goal is to recommend a set of physical activities *A* = {*a*_1_*, a*_2_*,* *…, a_m_*} based on contextual weather conditions while ensuring the weekly activity goal is met.

We define the following constraints:

- Constraint 1: Activity Duration. Each recommended activity must contribute to achieving a total weekly activity time *T*_weekly_ ≥ *T*_goal_, where *T*_goal_ is the target duration specified by the user or guidelines.
  $$\sum_{i=1}^{m}d(a_{i})\;\cdot \;p(a_{i})\;\;\geq \;\;T_{goal}$$ (1)
  where *d*(*a_i_*) is the duration of activity *a_i_* and *p*(*a_i_*) ∈ {0*,* 1} indicates whether *a_i_* is recommended (1) or not (0).
- Constraint 2: Weather Suitability. Activities must align with the current and forecasted weather conditions *x _j_*. For instance:
  $$\mathrm{If}\;\;x_{j}\;\;=\;\;\mathrm{rainy},\;\;\mathrm{then}\;\;p(a_{i})\;\;=\;\;0\;\;\forall a_{i\;\;}\in \;A_{outdoor}$$ (2)
  where *A*_outdoor_ is the subset of outdoor activities.
- Constraint 3: User Preferences. The set of recommended activities must consider user preferences *P*, such as time of day or type of activity.
  $$\mathrm{If}\;u_{i}\;\in P,\;\;then\;\;p(a_{i})\;\;=\;\;1$$ (3)
  where *u_i_* is a user-preferred activity. The optimization objective is:
  $$\max\sum_{i=1}^{m}[w_{i}\;\cdot \;\;p(a_{i})]$$ (4)
  where *w_i_* is the weight indicating the priority or benefit of recommending activity *a_i_*.

#### 2.3.2 Problem classification

To classify the problem, we analyze its relationship to well-known computational problems. The recommendation problem can be compared to the *knapsack problem*, where the goal is to maximize the cumulative benefit of actions (analogous to the value of items) under constraints such as time and weather (analogous to weight limits). The knapsack problem is NP-complete. The associated decision problem is to determine whether there exists a subset of activities *A*^′^ ⊆ *A* such that:

$$\sum_{a_{i}\in A^{\prime }}d(a_{i})\;\;\geq \;\;T_{goal}$$

In terms of classification, the problem is in **NP** because given a solution (a set of actions), we can verify its feasibility in polynomial time by checking constraints. The problem is **NP-hard** because it involves the complexity of the knapsack problem when generalized to many constraints, such as weather and user preferences. Furthermore, the problem is **NP-complete** because it is both in NP and as hard as any problem in NP.

## 3 Methods

**Figure 5:**
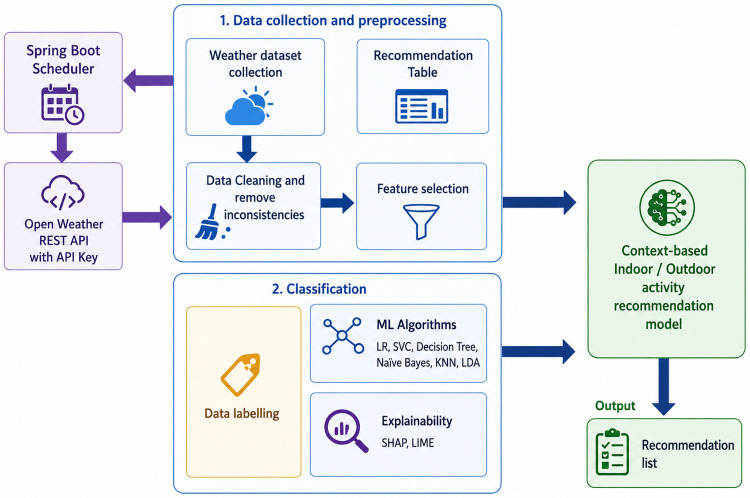
The adopted approach for data collection, data processing, and recommendation generation.

**Figure 6:**
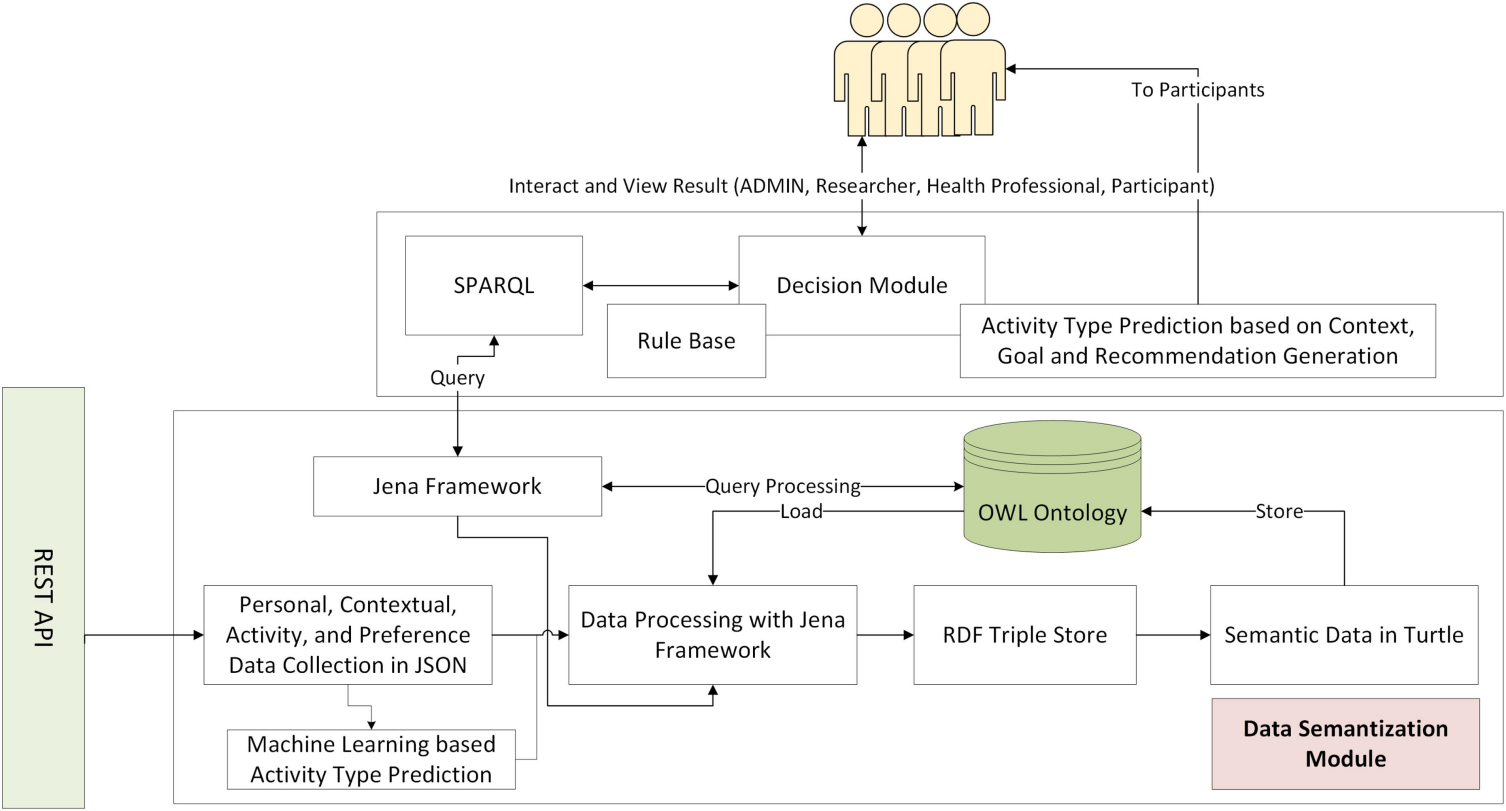
The designed semantic module for data annotation in eCoaching.

This section describes the adopted methodology for the study. Here, we describe the data collection process, considered ML classification models and their explanation models, model evaluation metrics, and set up for experimentation. The adopted approach from data collection to recommendation generation has been depicted in Figure 5, and the semantic module for data annotation is presented in Figure 6. Moreover, to test the activity eCoach system based on weather data, we have introduced different test cases to cover various scenarios to ensure its effectiveness and accuracy. Table 2 consolidates key symbols and notations, offering a quick reference.

**Table 2:** Symbols and notations used in the paper.

Symbol/Notation	Description
*T*	Timestamp of collected data
*X*	Set of input features
*y*	Class label for classification
*O*	Ontology
*C*	Set of concepts in the Ontology
*R*	Set of relationships in the Ontology
*TP*	True Positive in confusion matrix
*FP*	False Positive in confusion matrix
*TN*	True Negative in confusion matrix
*FN*	False Negative in confusion matrix
*Accuracy*	Classification accuracy
*Precision*	Precision metric for classification
*Recall*	Recall metric for classification
*F*1-score	F1-score metric for classification
*MCC*	Matthews Correlation Coefficient
*W* (*t*)	Weather data at time *t*
*S*(*t*)	Individual daily activity status at time *t*
*P*	Set of personal preferences
*DueCo*	Duration of eCoaching
*Δ_i_*	Contextual recommendation components (*i* = 1*,* 2*,* *…,* 7)
*µ*	Mean
*σ*	Standard deviation
*P*25*, P*50*, P*75	25th, 50th (median), and 75th percentiles
|*S*|	Size of dataset *S*

**Table 3:** The parameter list of the considered ML models for hyperparameter tuning.

Model	Parameters
LR	’penalty’: [’l1’, “l2’], ‘*α*’: [0.1, 1, 10], ’solver’: [’liblinear’, ‘saga’], ‘max_iter’: [100, 200, 500]
SVC (linear)	’*α*’: [0.1, 1, 2], ‘loss’: [’hinge’, ’squared_hinge’], ‘penalty’: [’l1’, ‘l2’], ‘max_iter’: [100, 200, 500, 1000]
SVC (RBF)	’*α*’: [0.1, 1, 2, 5], ‘gamma’: [0.1, 0.01, 0.001], ‘kernel’: [’rbf’]
DT	’criterion’: [’gini’, ‘entropy’], ‘max_leaf_nodes’: [2, 4, 6, 8, 10, 12], ‘min_samples_split’: [2, 3, 4]
NB	’var_smoothing’: [1e-9, 1e-8, 1e-7, 1e-6, 1e-5]
KNN	’n_neighbors’: [2, 4, 6], ‘weights’: [’uniform’, ‘distance’], ‘p’: [1, 2]
LDA	’solver’: [’svd’, ‘lsqr’, ‘eigen’], ‘shrinkage’: [None, ‘auto’, 0.1, 0.5, 1.0], ‘tol’: [0.0001, 0.001, 0.01]

### 3.1 Methodological novelty

The methodological novelty of this work lies not in the introduction of new machine learning algorithms, but in the principled integration of explainable predictive modeling with semantic knowledge representation. This design enables the translation of probabilistic predictions into logically consistent, human-interpretable recommendations, addressing a critical gap in current eHealth and eCoaching systems.

### 3.2 Data collection and data description

Data collection refers to the process of gathering information or data from various sources for analysis and research. The process may involve collecting quantitative or qualitative data, or a combination of both. Data collection can be manual, such as through paper-based surveys, or electronic, such as through online surveys or electronic health records. Data can also be collected passively, for example through sensors, posts in social media, web traffic, and Web API. Once data is collected, it needs to be cleaned, organized, and analyzed to generate insights and draw conclusions. Effective data collection is critical to making informed decisions and improving processes, products, and services. In this study, we have collected external weather data OpenWeatherMap REST API against API Key validation, based on the geographic location of the city (e.g., latitude and longitude). REST APIs use HTTP requests to get or send data, making them a common choice for data collection with the following steps−identify the API endpoint, understand the API documentation, make an HTTP request, receive the API response, and parse and process the data. The collected dataset is already clean and without anomalies. We annotated the collected weather data with Ontology as described in the previous section for semantic annotation. OpenWeatherMap (14) is a popular API that provides current and forecast weather data around the world. It is a RESTful API that allows developers to retrieve weather data in various formats (33), including JSON, XML, and HTML. In order to use the OpenWeatherMap API, we register for an API key, which we obtain by creating an account on the OpenWeatherMap website. To get weather data for a specific location, we provide the latitude and longitude of that location in the API request. Here's an example of how we retrieved current weather data for Kristiansand in (34). This request (34) returns weather data in JSON format. We used this data to see current weather conditions such as temperature, humidity, and wind speed. Moreover, OpenWeatherMap offers the following range of weather information −

- Current weather conditions: We can retrieve information on the current temperature, humidity, wind speed and direction, atmospheric pressure, cloud cover, and precipitation for a specific location.
- Forecast data: OpenWeatherMap provides weather forecast data for up to 16 days in advance, including hourly and daily forecasts. This includes information on the expected temperature range, wind speed and direction, precipitation, and cloud cover.
- Historical weather data: OpenWeatherMap offers historical weather data for locations around the world, which includes information on past temperatures, wind speeds, and precipitation.
- Air quality data: OpenWeatherMap provides real-time air quality data for locations around the world, including information on pollutants such as particulate matter, nitrogen dioxide, and ozone.
- Weather maps: OpenWeatherMap offers a range of weather maps that display current and forecast weather conditions, including temperature, precipitation, cloud cover, and wind speed and direction.

In this context, the Weather forecast data are highly valuable for activity recommendations, as it provides crucial information about the expected weather conditions in a specific location and time period. By incorporating weather forecast data into activity recommendations, we tried to formulate more personalized and context-aware suggestions for users. We developed a scheduler module using the Java Spring-boot framework to periodically (i.e., every 30 min) collect current and forecast data of *thirteen cities in southern Norway from the OpenWeatherMap API* and store them in a relational database for persistence. We chose cities from the Southern-Norway due to their unique and prominent weather patterns. The duration of data collection was from 7/6/2021 to 12/12/2022. It produced a total 3,37,624 number of records (58.1 Megabytes in size). The data were cleaned further to annotate them with the designed and developed Ontology model. We stored the Ontology models with instances in a tuple database (TDB) for querying and reasoning. The collected features of the dataset are−timestamp, city, country, code, status, description, temperature, real feel, minimum temperature, maximum temperature, pressure, humidity, sea level, ground level, visibility, wind speed, wind degree, wind gust, and overall clouds. We encoded the categorical features with an integer value. The description of the collected data has been elaborated in the API documentation under the *Current and Forecast weather data of OpenWeatherMap collection (*14, 35). The collected dataset primarily represents Nordic and temperate seasonal weather patterns observed in Southern Norway. Consequently, the learned activity-type classification patterns may not directly generalize to tropical, continental, arid, or Mediterranean climate regions without additional retraining or adaptation. Therefore, the present work should be interpreted as a proof-of-concept framework validated under Nordic environmental conditions.

#### 3.2.1 Data quality and temporal coverage analysis

To ensure robustness, the collected weather dataset was analyzed for temporal continuity, seasonal coverage, and missing-value patterns. The dataset spans 18 months across four meteorological seasons, enabling the model to capture seasonal variability and extreme weather transitions. Statistical summaries (mean, variance, and interquartile ranges) were computed for all continuous features to verify distributional stability prior to modeling.

### 3.3 Feature engineering and data labeling

Statistical methods for feature selection can facilitate the efficiency, accuracy, and interpretation of machine learning models, this makes it easier to utilize complex datasets and extract meaningful information from the data. Therefore, we used the following statistical feature selection methods (36, 37)−Select-K-Best, Extra Tree Classifier, Recursive Feature Elimination, Principal Component Analysis, and correlation analysis. We excluded features with a correlation coefficient of ≥ 0.8. We eliminated strongly connected features and retained features based on their fitness score or ranking. A quantile analysis helped to remove outliers in the dataset based on the distribution of data [e.g., min, Q1 (25%), median, Q3 (75%), max, and IQR (Q3-Q1)]. Based on the features associated with weather data, we classified the dataset into the following two classes: indoor activity (0) and outdoor activity (1). The activity-type labeling strategy was formulated based on environmental suitability principles discussed in prior studies on weather-sensitive physical activity behavior (9, 10, 12). Previous research has shown that adverse weather conditions such as heavy rain, snow, high wind speed, and low visibility reduce outdoor activity participation and increase preference toward safer indoor alternatives. The classification rules are as follows and are based on weather descriptions for indoor and outdoor activities −

- Outdoor activity (1) if (description == “light rain’ or “overcast clouds’ or “light intensity drizzle” or “light snow” or ’scattered clouds’ or “few clouds” or “mist” or “clear sky” or “light rain and snow” or “light shower snow”)
- Indoor activity (0) if (description == “broken clouds” or “drizzle” or “moderate rain” or ’snow' or “fog” or “drizzle rain” or ’shower snow' or “heavy intensity rain” or “thunderstorm with rain” or “very heavy rain” or ’shower rain' or “heavy intensity drizzle” or “thunderstorm” or ’shower sleet' or ’sleet’ or “rain and snow” or “light shower sleet” or “heavy snow”)

#### 3.3.4 Feature relevance and statistical significance

Feature relevance was assessed using a combination of correlation analysis, ANOVA tests, and model-based importance ranking. Features exhibiting multicollinearity (correlation coefficient ≥ 0*.*8) were excluded to reduce redundancy and improve generalization. Statistical hypothesis testing confirmed that all retained features had a significant influence (*p* *<* 0*.*05) on activity type classification.

### 3.4 Machine learning classification models

We used standard ML algorithms for classification with 5-fold cross-validation, random state=42, and Grid-search for hyperparameter optimization as they represent most common families of supervised ML algorithms and work well with medium size dataset for binary classification (38–41) Logistic Regression (LR), Support Vector Classifier (SVC), Decision Tree (DTree) Classifier, Naïve Bayes, K-Nearest Neighbor (KNN), and Linear Discrimination Analysis (LDA). *Logistic regression* is a statistical technique that models the probability of a dependent variable that is categorical or binary, based on one or more independent variables. It's effective for linearly separable data and is simple to comprehend, which makes it a popular choice for a variety of classification problems. The *SVC* is another popular algorithm for classification. It's derived from the concept of finding the hyperplane that best separates the data into two classes. SVC is effective in conjunction with both linearly separable and non-linearly separable data. The *Decision Tree* Classifier is a popular algorithm that creates a Decision Tree by recursively partitioning the data based on the most significant attribute. The Decision Tree can be employed to predict the class of a new data point by following the path from the root to the leaf node. Decision Tree is simple to comprehend and interpret, which makes them beneficial for a variety of classification issues. *Naïve Bayes* is a probabilistic algorithm that assumes that the significance of a certain attribute in a class is independent of the presence of other attributes. It's straightforward, fast, and can work with high-dimensional data. *K-Nearest Neighbor* is a non-parametric algorithm that predicts the class of new data based on the classes of its K-nearest neighbors in the training dataset. It's a simple and effective algorithm for small datasets; however, can be computationally intensive for large datasets. *LDA* is an unsupervised classification algorithm that attempts to find the linear combination of attributes that best separates the classes. Instead of modeling the probability of the dependent variable, it models the actual boundary between the classes directly. The selection of the algorithm chosen is highly dependent on the nature of the problem, the size of the dataset, the data type, and the desired performance. It's often beneficial to experiment with multiple algorithms and compare their effectiveness for a particular problem. However, for binary classification on a medium-sized dataset, Logistic Regression, SVC, and Decision Tree classifiers are typically good choices as of their simplicity, transparency, and capacity to deal with nonlinear data. The adopted steps for the machine learning classification are mentioned in Algorithm 2.

Algorithm 2 **Machine learning classification.**

1: **Input**: Training dataset *D*, Test instances *X*_test_

2: **Output**: Predicted class labels for *X*_test_

3: **Preprocessing**:

4: Clean and preprocess the training dataset *D*

5: **Model Training**:

6: Select a classification algorithm *A*

7: Set algorithm parameters and hyperparameters

8: Train the classifier model *M* using *D*

9: **Prediction**:

10: **for** each instance *x* in *X*_test_ **do**

11:  Predict the class label *y* for *x* using the trained model *M*

12:  Assign *y* as the predicted label for *x*

13: **return** Predicted class labels for *X*_test_

Overall, the decision tree classifier produced the best results on the selected classification problem. For classification, we used the following approach−loading of the labeled dataset, split the dataset into dependent and independent features, building a classification model to train the model against an optimal number of features, evaluating a given model with five-fold cross-validation and scoring=“accuracy”, hyperparameter tuning with the grid-search method, and compare the performances of the selected classification models. A Decision Tree is a supervised learning algorithm used in machine learning and data mining (42, 43). It is a graphical representation of all possible solutions to a decision based on a set of conditions or attributes. A Decision tree consists of nodes and branches, each node represents a decision or property test, and each branch represents the outcome of that decision. The root node represents the initial decision, while the leaf nodes represent the final result or decision. Construction of a Decision Tree involves selecting an attribute that best divides the data into two or more subsets based on criteria such as information gain, gain ratio, or Gini index (42, 43). This process is repeated recursively for each subset until a stopping criterion is met, such as when all instances in a subset belong to the same class, or when there are no more attributes to split on. Here are the adopted steps to build a decision tree classification model −

- Selection of labeled dataset.
- Selection of an attribute to split the data based on some criterion such as information gain, gain ratio, or Gini index.
- Divide the data to be classified into subsets based on the values of the selected attribute (with 5-fold cross-validation).
- Repeat steps 1–3 recursively on each subset until a stopping criterion is met, such as when all instances in a subset belong to the same class or when there are no more attributes to split on.
- Assignment of a class label to each leaf node based on the majority class in the corresponding subset.
- Use the decision tree classification model to classify new instances by following the path from the root node to the appropriate leaf node based on the values of the attributes.
- Store the model as a pickle file.
- Load the model, train it with growing weather data, and store it back as a pickle file.

The mathematical representation of a decision tree classifier is as follows−given a training set D = (*x*_1_,*y*_1_), (*x*_2_,*y*_2_), .., (*x_n_*,*y_n_*) where *x_i_* is a vector of input attributes and *y_i_* is the corresponding class label, we want to learn a decision tree that can predict the class label for new instances. Let X be the set of input attributes and Y be the set of possible class labels. We can represent the Decision Tree as a binary tree where each internal node represents a decision on an attribute and each leaf node represents a class label. Let *T* be the Decision Tree and *T_i_* be a subtree rooted at node *i*. We can define the entropy of a set S with respect to class labels Y as follows:

$$\mathrm{Entropy}(S)\;=\;\sum_{y\in Y}\;p(y)\log_{2\;}\;p(y)$$ (6)

where *p*(*y*) is the proportion of instances in *S* that belong to class *y*. We can use entropy to measure the impurity or randomness of a set of instances with respect to their class labels. The goal of the Decision Tree algorithm is to find the attribute that minimizes the entropy of the subsets created by the splitting criterion. Let A be an attribute and V be a set of possible values for A. We can define the information gain of A with respect to a set S as follows:

$$InformationGain(A,S)=\;Entropy(S)-\;\sum_{V_{i}\in V}\frac{\left|S_{i}\right|}{\left|S\right|}\;*Entropy(S_{i})$$ (7)

where *S_i_* is the subset of *S* where the value of *A* is *V_i_*, and |*S_i_*| and |*S*| are the sizes of *S_i_* and *S*, respectively. The attribute with the highest information gain is selected as the split criterion, and the data is divided into subsets according to the value of the attribute. This process is repeated recursively until the termination condition is met. Finally, to classify a new instance x, we traverse the decision tree from the root to the corresponding leaf node according to its attribute value and assign the class label of the leaf node as the predicted class label of x. There are variants of this mathematical model that use other split criteria, such as gain ratio and Gini index. The model can also be extended to handle regression tasks by predicting continuous values instead of class labels. The Decision Tree Classification has been elaborated in Algorithm 3.

Algorithm 3 **Decision tree classification.**

**Require:** Training data, Test data

**Ensure:** Predicted classes for Test data

 1: **Initialization:** Split data into features and labels

 2: **Model Training:** Train a decision tree classifier using training data

3: **Model Testing:** Test the classifier using test data

4: **Prediction:** Predict classes for test data using the decision tree model

The time complexity analysis of decision tree classification involves two major phases,−a. The construction phase involves recursively partitioning the dataset based on the selected features. For each internal node (decision node) in the tree, the algorithm evaluates the best split based on a criterion (e.g., Gini impurity, entropy) among all features. This operation requires examining all possible features for each node. Let *n* be the number of samples in the dataset, and *d* be the number of features. The worst-case time complexity of finding the best split for each node is *O*(*n* · *d*), as it requires evaluating each feature for each sample to determine the best split. The decision tree construction process continues until all samples belong to the same class, or when there are no more features left to split on. The depth of the decision tree is at most *d* (the number of features). Therefore, the overall time complexity of the decision tree construction phase is *O*(*n* · *d*^2^) in the worst case, and b. During the prediction phase, an input instance traverses the decision tree from the root node to a leaf node based on the feature values of the instance. The time complexity of the prediction phase is proportional to the depth of the decision tree. In the worst case, when the decision tree is highly unbalanced, the depth can be *d*. Therefore, the time complexity of the prediction phase is *O*(*d*).

#### 3.4.1 Hyperparameter tuning

To address the need for additional details on hyperparameter tuning and model configuration selection, we employed a comprehensive approach using **GridSearchCV** to optimize model hyperparameters (44, 45). GridSearchCV performs an exhaustive search over a predefined parameter space, testing combinations to identify the best configuration based on validation set performance. The objective was to tune hyperparameters such as the learning rate *α*, maximum depth of decision trees, kernel types in support vector classifiers, and the number of neighbors in K-Nearest Neighbor models. These parameters were selected for their significant impact on model learning dynamics and classification accuracy, crucial for managing the complex structure of physical activity datasets. This tuning process allowed us to balance predictive accuracy with model robustness, ensuring optimal configurations for both balanced and imbalanced datasets. The chosen configurations achieved a high degree of accuracy, F1-score, and MCC, providing reliable performance across varied data distributions. The adopted hyperparameter tuning process has been elaborated in Box 1.

BOX 1 Adopted hyperparameter tuning process.

Hyperparameter Tuning Process [hbt]

- **Hyperparameter Grid Definition**: For each model, we defined a grid of parameters (see Table 3) including:
  − *Learning rate* (*α*) for gradient-based models (e.g., logistic regression),
  − Max depth, *criterion*, and *split parameters* for decision trees,
  − *Kernel types* and *regularization* for support vector classifiers,
  − Number of neighbors and *distance metrics* for K-Nearest Neighbor.
- Parameter Space Exploration: Using GridSearchCV, all parameter combinations were evaluated based on performance metrics on the validation set.
- 5-Fold Cross-Validation: We employed stratified K-fold cross-validation to preserve class distribution in each fold, critical for imbalanced data, thus enhancing model robustness and generalizability.
- Execution Across Iterations: Each model was trained and validated five times with shuffled datasets, averaging performance scores to mitigate variance across data splits.
- Final Model Selection Criteria: The final hyperparameter configuration for each model was selected based on:
  – High average scores on accuracy, precision, recall, F1-score, and Mathew's Correlation Coefficient (MCC),
  – Model simplicity, favoring lower complexity configurations (e.g., reduced depth in decision trees or fewer neighbors in KNN) if performance was comparable, to reduce overfitting risks and improve interpretability.

#### 3.4.2 Classification explanation models

Model interpretability focuses on providing understandable explanations of how a model works and makes predictions, while model interpretability focuses on understanding the inner workings of the model itself (46). Both concepts are important in machine learning because they help users understand and trust the models they are using and can improve accuracy and performance. Nowadays, it is not enough to train an accurate model to use it successfully. We should also be able to explain the model, i.e., explain its behavior, the features that contribute to a given prediction, and the important attributes overall. Tree explanations are intuitive and straightforward because the decision process is easily visualized through the tree's structure. By following the path from the root node to the corresponding leaf node, we can see which features are most relevant for the prediction, making decision trees valuable for understanding how the model makes its decisions. While decision tree explanations can be valuable for understanding individual model behavior and feature importance, LIME addresses the limitations of decision trees in handling complex models, and high-dimensional data and providing more nuanced and locally interpretable explanations. Ultimately, the choice between LIME and decision tree explanations depends on the specific use case, the model's complexity, and the level of interpretability required. We used the LIME Model (47) on top of the Decision Tree Classifier to explain the feature importance for classification. The primary function of LIME is to create a simple, yet comprehensible model, such as a linear regression or decision tree, that describes the behavior of the complex black-box model near its maximum (47). The explanations are derived from the interpretable model by fitting it to a portion of the original data that is considered the “neighborhood” or “perturbed” instances of the object to be explained. LIME generates explanations that are represented by the value of importance associated with the features, this value indicates the significance of each feature to the prediction of the instance in question (47). These values of significance are derived from the degree to which the instances are perturbed in the interpretable model. LIME is model agnostic and performs local interpretation to explain single predictions of a classification model (47). LIME builds a sparse linear model around each prediction to explain how the black-box model works in that local environment (47). The LIME algorithm has been expressed in Algorithm 4.

Algorithm 4 **LIME algorithm.**

1: Select an instance to explain

2: Generate perturbations around the instance

3: Predict the output for perturbed instances

4: Build a local linear model to approximate the behavior

5: Assign weights based on proximity to the instance

6: Obtain feature weights as explanations

Table 4 presents the workflow of the explainable artificial intelligence module using the LIME framework. The pro­cess consists of perturbation generation, prediction estimation, local surrogate modeling, feature contribution analysis, and visualization-based interpretation to explain the predictions generated by the Decision Tree classifier.

**Table 4:** Workflow of the X-AI explanation process using LIME.

Phase	XAI Component	Description	Output
Phase 1	Perturbation Generation	Generate synthetic neighboring samples around an instance	Local neighborhood data
Phase 2	Prediction Estimation	Predict probabilities using trained Decision Tree model	Prediction probabilities
Phase 3	Local Surrogate Modeling	Train interpretable linear approximation model	Local explanation model
Phase 4	Feature Contribution Analysis	Estimate positive and negative feature contributions	Feature importance scores
Phase 5	Visualization and Interpretation	Visualize explanation probabilities and feature weights	Human-interpretable explanation

#### 3.4.3 Classifier evaluation metrics

The performance of machine learning-based binary-class classifiers had been evaluated against well-known discrimination analysis. Multiple well-established metrics, such as classification report, confusion matrix, precision, recall, specificity, accuracy score, and F1 score had been estimated (2). A confusion matrix is a 2-D table (*actual* vs. *predicted*) and both dimensions have four options, namely, *true positives (TP)*, *false positives (FP)*, *true negatives (TN)*, and *false negatives (FN)*. *TP* is an outcome where the model estimates the positive class accurately; *TN* is an outcome in which the model correctly predicts the negative class; *FP* is an outcome where the model estimates the positive class inappropriately; and *FN* is an outcome in which the model predicts the negative class incorrectly. The respective equations are −

$$Precision=\;\frac{TP}{TP+\;FP}$$ (8)

$$Recall=Sensistivity=\;\frac{TP}{TP+FN}$$ (9)

$$Accuracy=\;\frac{TP\;+\;TN}{TP\;+\;TN\;+\;FP\;+\;FN}$$ (10)

$$F-score=\frac{2\;\times \;Recall\times Precision}{Recall+Precision}$$ (11)

When the labels are imbalanced, accuracy is not the best metric to use. As an illustration, let's say that 75% of the instances come from class 1, and 25% comes from class 0. If one consistently predicts the majority class label, which in this case would be class 1, one can easily get 75% accuracy. A higher value from the above expressions represents a better performance of a model, and this applies to all performance metrics. In contrast, *bias* is an error due to erroneous assumptions in the learning algorithm, and *variance* is an error from sensitivity to small fluctuations in the training set. While high bias leads to under-fitting, high variance results in overfitting. *Accuracy* and *F1-scores* can be confusing as it does not fully account for the sizes of the four categories of the confusion matrix (TP, FP, TN, and FN) in the final score calculation. In comparison, the *MCC* is more informative than the *F1-score* and *Accuracy* because it considers the balanced ratios of the four confusion matrix categories (i.e., *TP, TN, FP*, and *FN*). The *F1-score* depends on which class is defined as a positive class. However, *MCC* does not depend on which class is the positive class, and it has an advantage over the *F1-score* as it keeps away from incorrectly defining the positive classes (48). The *MCC* is represented as follows (23).

$$MCC=\frac{TP\times TN-FP\times FN}{\sqrt{\left(TP+FP)(TP+FN)(TN+FP)(TN+FN\right)}}$$ (12)

#### 3.4.4 Model robustness and overfitting analysis

Model robustness has been evaluated using stratified five-fold cross-validation and scalability testing with increasing sample sizes. Training and test accuracy curves have been analyzed to detect overfitting. The minimal divergence between training and testing performance indicates stable generalization across varying data volumes.

#### 3.4.5 Ontology evaluation

Our designed and developed ontology model is evaluated using the following two metrics, inference time and query execution time. Protégé provides a list of inference engines (49) such as HermiT, Fact++, Pellet, KAON2, and RacerPro to check logical and structural consistency. We compared the average thinking times and selected the best thinkers for our ontology. Moreover, we recorded the execution time of SPARQL queries in Protégé. We load ontology files into the Jena Fuseki server in “TTL” format to validate them at SPARQL query execution time. We use the Apache Jena Framework to query each ontology class, predicate, topic, and object.

### 3.5 Machine learning pipeline design

A well-designed machine learning pipeline streamlines the development process, ensures data quality, enables model selection and optimization, and facilitates the deployment and maintenance of machine learning models. It improves the efficiency and effectiveness of machine learning projects, leading to better models and successful applications in various domains. A typical machine learning pathway has multiple consecutive steps that have different purposes during the entire process. The design of the pipeline has been versatile enough to deal with alterations and enhancements throughout the process. Here is the common procedure involved in a machine learning pipeline −

- Data Collection: We collected external weather datasets from different cities of Southern Norway through OpenWeath-erMap API following the GDPR guidelines, and no identity has been disclosed.
- Data Cleaning and Preprocessing: We checked if any missing, anomalous, or NaN data values. We encoded categorical features, if any.
- Feature Engineering: We used standard statistical techniques for feature optimization to create an optimal feature set.
- Data Splitting: We used a ratio of 60:20:20 with shuffling, randomization, and stratification techniques to train, validate, and test ML models.
- Model Selection: We used traditional ML classifiers and selected the best classifier based on the metrics, such as accuracy, F1-score, precision, recall, MCC, and mean and standard deviation of the accuracies against cross-validation.
- Model Training: We used the Stratified K-Fold method and 5-fold cross-validation (Grid-Search Classifier CV) techniques.
- Model Evaluation: We compared and analyzed the individual ML model's scalability.
- Hyperparameter Tuning: We used the traditional Grid-Search method for hyperparameter training.
- Model Validation: We used the 5-fold cross-validation (Grid-Search Classifier CV) techniques.
- Model Deployment: We prepared a pickle file to store the best-performing trained classifier models for the imbalanced and balanced datasets.
- Monitoring and Maintenance: We executed all the above steps in an incremental fashion to allow the classification of real-time growing weather data.

Overall, Algorithm 5 describes the adopted and summarized steps to build our machine learning classification pipeline.

Algorithm 6 informs the high-level steps for hyperparameter tuning in machine learning classification.

Algorithm 5 **Machine learning classification pipeline with model deployment.**

**Require:** Training data, Test data

**Ensure:** Predicted classes for Test data

 1: **Initialization:** Split data into features and labels, Define classifier, Set hyperparameters

2: **Preprocessing:** Normalize/Standardize features, Handle missing values

3: **Model Training:** Train the classifier using training data

4: **Model Testing:** Test the classifier using test data

5: **Prediction:** Predict classes for test data

6: **Model Deployment:** Deploy the trained model for real-world use

Algorithm 6 **Hyperparameter tuning for machine learning classification.**

**Require:** Training data, Hyperparameter search space

**Ensure:** The best hyperparameters for the classifier

 1: **Initialization:** Split data into features and labels, Define classifier

2: **Preprocessing:** Normalize/Standardize features, Handle missing values

**3:** Hyperparameter Tuning:

4: **for** each combination of hyperparameters in the search space **do**

5: Train the classifier using training data with current hyperparameters

6: Evaluate the classifier using cross-validation

7: Keep track of the hyperparameters with the best performance

8: **Best Hyperparameters:** Use the hyperparameters with the best performance

9: **Model Training:** Retrain the classifier using the best hyperparameters and the full training data

10: **Model Testing:** Test the classifier using test data

11: **Prediction:** Predict classes for test data

12: **Model Deployment:** Deploy the trained model for real-world use

### 3.6 Test case design for system verification

By testing the activity eCoach recommendation system with various weather scenarios, user preferences, and real-time updates, we can ensure that the system is robust, adaptable, and capable of providing relevant and practical activity suggestions based on weather data. Here are some sample test cases we have considered in the Supplementary Material S3. The test cases help in verifying that the system can handle different real-world situations and deliver valuable recommendations to users in a wide range of scenarios.

## 4 Results

This section describes the experimental setup, overall results obtained from the dataset, an explanation of the classification model based on feature importance, and Ontology verification. All experiments have been performed in accordance with relevant ethical guidelines and regulations as included in the Ethics approval and consent to participate subsection under the Declarations section.

### 4.1 Experimental setup

We used Colab or Google Colaboratory, a cloud-based Jupyter Notebook environment provided by Google, to run the experiment. The amount of memory available in Google Colab depends on whether you are using the CPU or GPU run-time and the exact hardware configuration you choose. The amount of storage required for a dataset depends on various factors, such as The size of the dataset, the number of features, and the data type. Typically, datasets that fit into the available memory can be efficiently loaded and processed in Colab. The amount of RAM provided by Google Colab (12 GB) can handle medium-sized datasets for most data science and machine learning tasks. Therefore, we used CPU instead of GPU for our ML classification tasks on a medium size dataset.

### 4.2 Experimental results

The experimental results are described as follows -

#### 4.2.1 Feature selection

**Figure 7:**
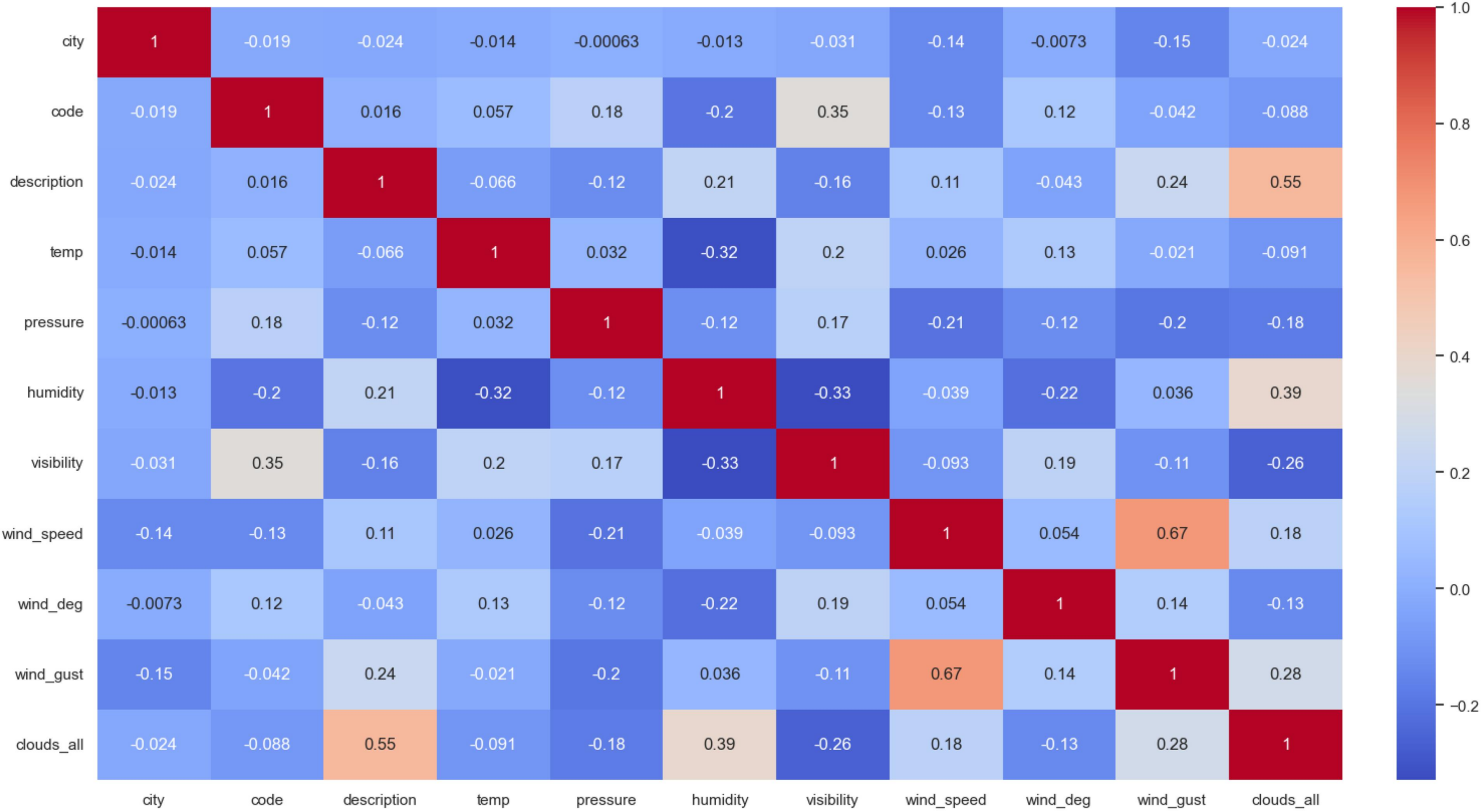
Correlation matrix of the selected weather features, showing the strength and direction of relationships between variables used for activity type classification.

**Figure 8:**
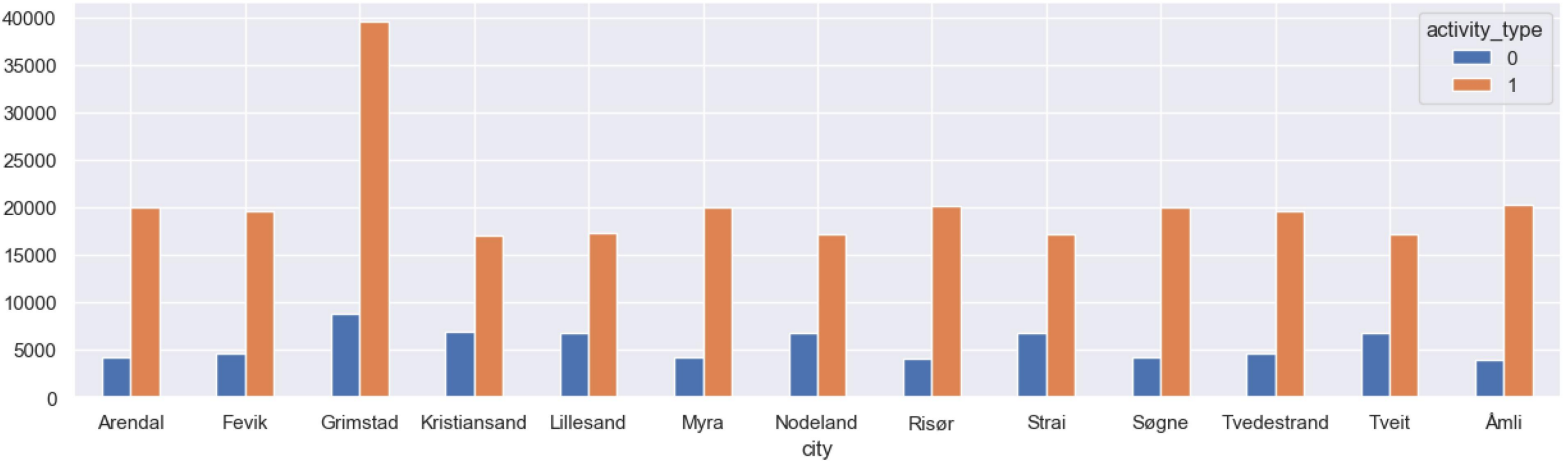
Distribution of indoor and outdoor activity types across different cities based on weather conditions, highlighting the variation in activity recommendations.

The feature analysis on the weather dataset excluded the following features with strong dependency−status, real-feel, temp min, and temp max. The resultant feature set consists of−city, code, description, temp, pressure, humidity, visibility, wind_speed, wind_deg, wind_gust, and clouds_all. The correlation matrix of the selected features has been depicted in Figure 7. The “description” feature helped in classifying activity types ('activity_type') for recommendations. We performed ANOVA typ=2 test on selected features against the activity_type feature with a hypothesis test−*p* *<* 0.05 means that the selected variable has a significant influence on the activity_type feature and *p* *>* 0.05 means that the selected variable has no significant influence on the activity_type feature. The ANOVA test results are described in Supplementary Materials S4, and all the *p*-values obtained as *<* 0.05. The distribution of activity types (outdoor:1 and indoor:0) for different cities has been depicted in Figure 8.

#### 4.2.2 Classification

**Figure 9:**
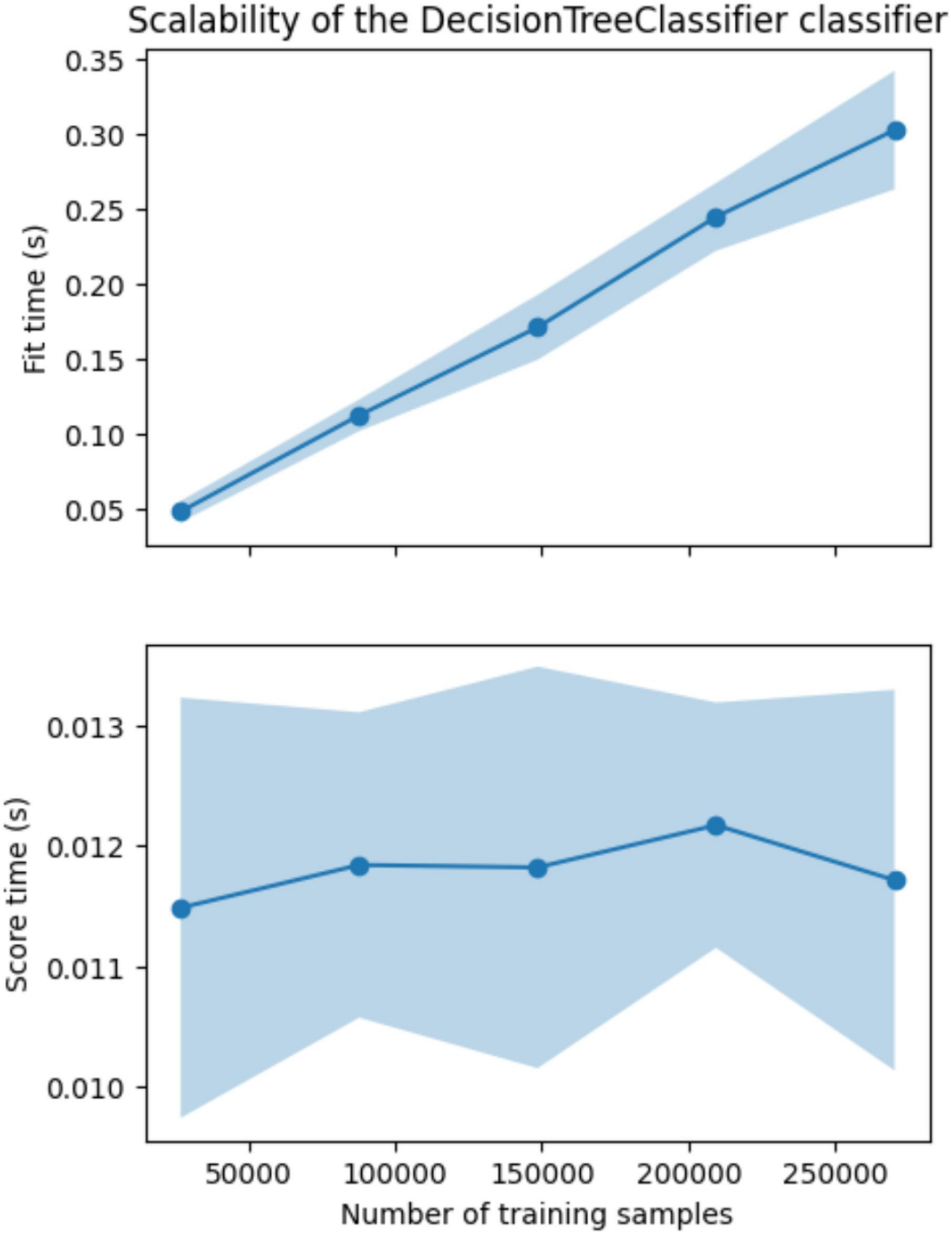
Scalability analysis of the decision tree classifier, depicting its performance as the number of data samples increases, ensuring robustness for larger datasets.

**Figure 10:**
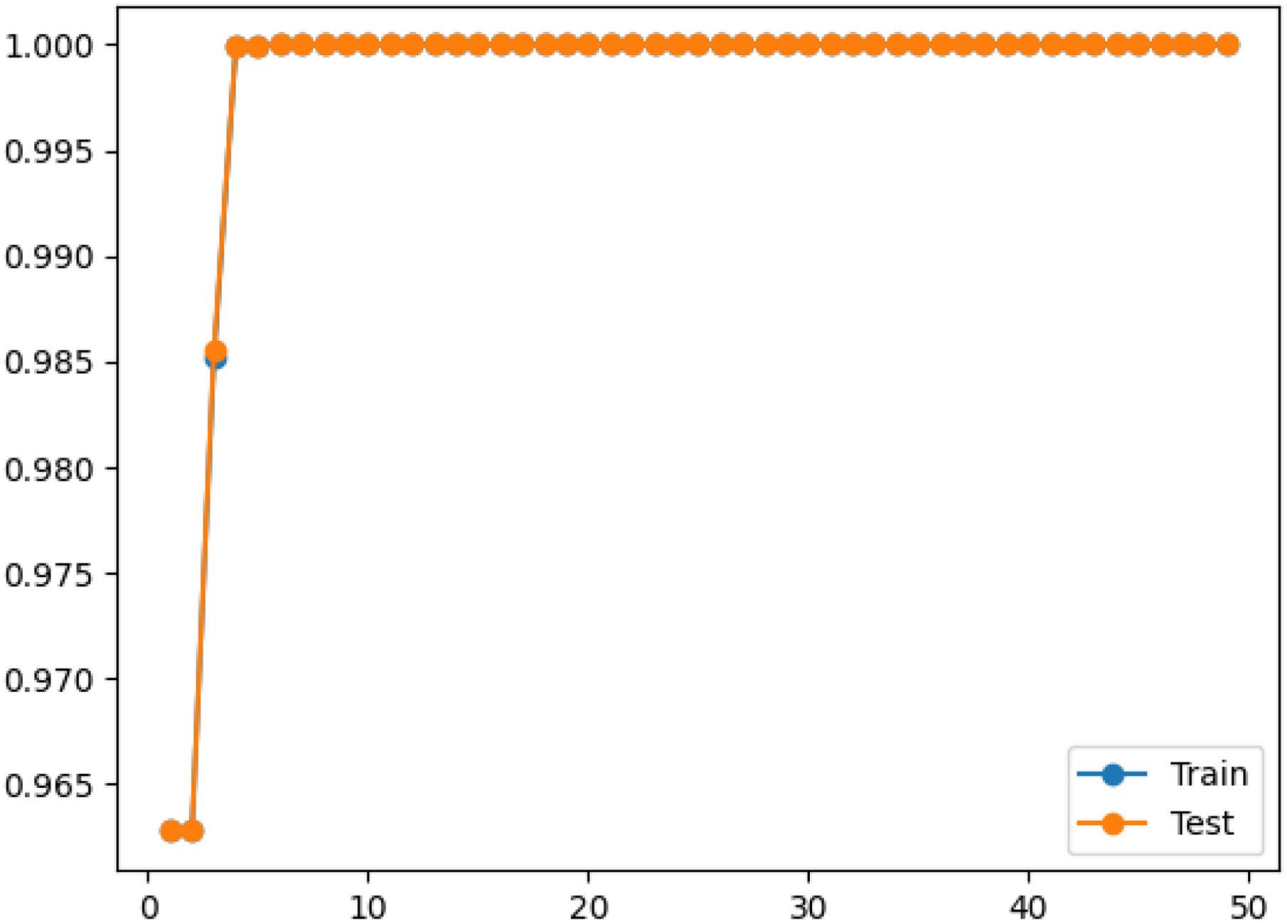
Train-test validation curves for the decision tree classifier, showing the accuracy of the model during training and testing phases to assess its generalization ability.

**Figure 11:**
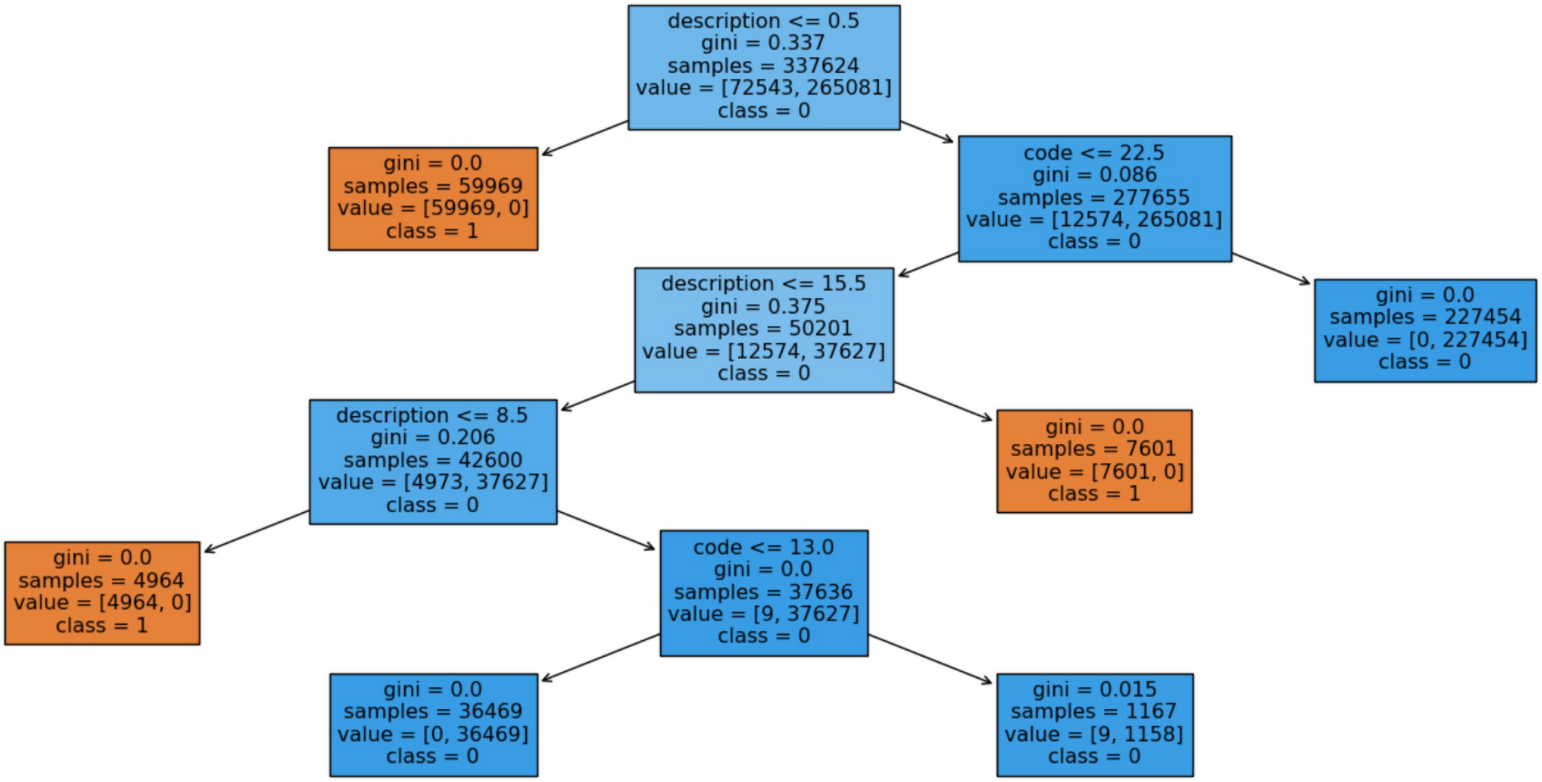
Decision tree visualization displaying the hierarchical structure of decision rules used for activity type classification, showing how weather features determine indoor or outdoor recommendations.

The Decision Tree Classifier with criterion="entropy” outperformed other activity type classification models (accuracy = 99.1%, F1-score=99.1%, precision=99.1%, recall=99.1%, and MCC=97.0%) with optimized hyperparameters as max_leaf_nodes=5, random_state=42, and min_samples_split=[2, 3, 4]. The criteria parameter determines the quality of the splits in the Decision tree. Entropy is one of the options for criteria parameters. Entropy is a measure of the impurity or disorder of a set of samples. The entropy criterion tries to minimize the entropy of the child nodes after splitting. The split with the lowest entropy is chosen because it results in the most homogeneous children. Entropy can be useful when we have categorical or continuous features that are not well separated, as entropy can still provide a meaningful measure of sample contamination. The activity type classification results are presented in Table 5 against different ML classifiers. We checked the scalability of the decision tree classifier with an increasing number of samples (see Figure 9) and performed the train-test validation (see Figure 10). The goal of scalability testing is to determine whether the decision tree model can handle large amounts of data and perform well under heavy workload conditions. The benefit of train-test validation is that it provides an estimate of the model's performance on new data, which is important for generalization. Moreover, we used the Graph viz tool for creating visualizations of the Decision Tree with maximum depth=5 (see Figure 11). The present study primarily focuses on contextual binary activity-type recommendation generation rather than ranked recommendation retrieval. Therefore, traditional recommendation ranking metrics such as NDCG, MRR, Precision@K, and Recall@K were not directly applicable to the current formulation. Tree explanations in Figure 11 with the decision tree involve understanding how the tree arrives at its predictions for individual instances, based on a series of rules applied to input features. The decision process follows the paths in the tree from the root node to a leaf node, with each internal node representing a decision based on a feature, and each leaf node providing the final prediction.

**Table 5:** Activity type classification results based on contextual data.

Models	Precision	Recall	F1-score	Accuracy	MCC
LR	97.0%	97.5%	98.0%	97.5%	93.5.0%
SVC (kernel=‘linear’)	94.0%	94.0%	94.0%	94.0%	91.0%
Decision Tree (criterion=‘entropy’)	99.1%	99.1%	99.1%	99.1%	97.0%
Decision Tree (criterion=‘gini’)	98.5%	98.5%	98.5%	98.5%	95.8%
Naive Bayes	96.0%	96.0%	96.0%	96.0%	92.0%
KNN (neighbou*r* = 2)	97.0%	97.0%	97.0%	97.0%	93.0%
KNN (neighbou*r* = 4)	96.0%	96.0%	96.0%	96.0%	92.5%
LDA	98.0%	98.0%	98.0%	98.0%	95.0%

Algorithm 7 **LIME for decision tree classification.**

**Require:** Black-box Decision Tree Classifier, Instance to be explained, Number of samples

**Ensure:** Local interpretable explanation for the prediction

 1: **Initialization:** Define a neighborhood around the instance to be explained

2: **Data Perturbation:** Generate random samples in the neighborhood

3: **Model Prediction:** Predict class probabilities for the perturbed samples using the black-box classifier

4: **Local Model Training:** Train a simple interpretable model (e.g., linear model) using the perturbed samples and their corresponding predictions

5: **Explanation Generation:** Use the local model coefficients as feature importance to explain the decision tree's prediction for the instance

In contrast, the LIME explanation offers several advantages over traditional tree explanations with decision trees, such as −a. it is a more versatile and widely applicable approach for explaining complex models, b. it explains individual predictions rather than the entire model, c. generates interpretable explanations by creating a simpler, locally interpretable model around the instance of interest, d. provides an estimate of uncertainty for each explanation and quantifies the reliability of the explanation, giving users an idea of how confident they can be in the provided explanation, e. is particularly useful for high-dimensional data, and f. helps users understand the factors contributing to a prediction in an understandable way. Decision tree explanations might not always offer such natural interpretability.

**Figure 12:**
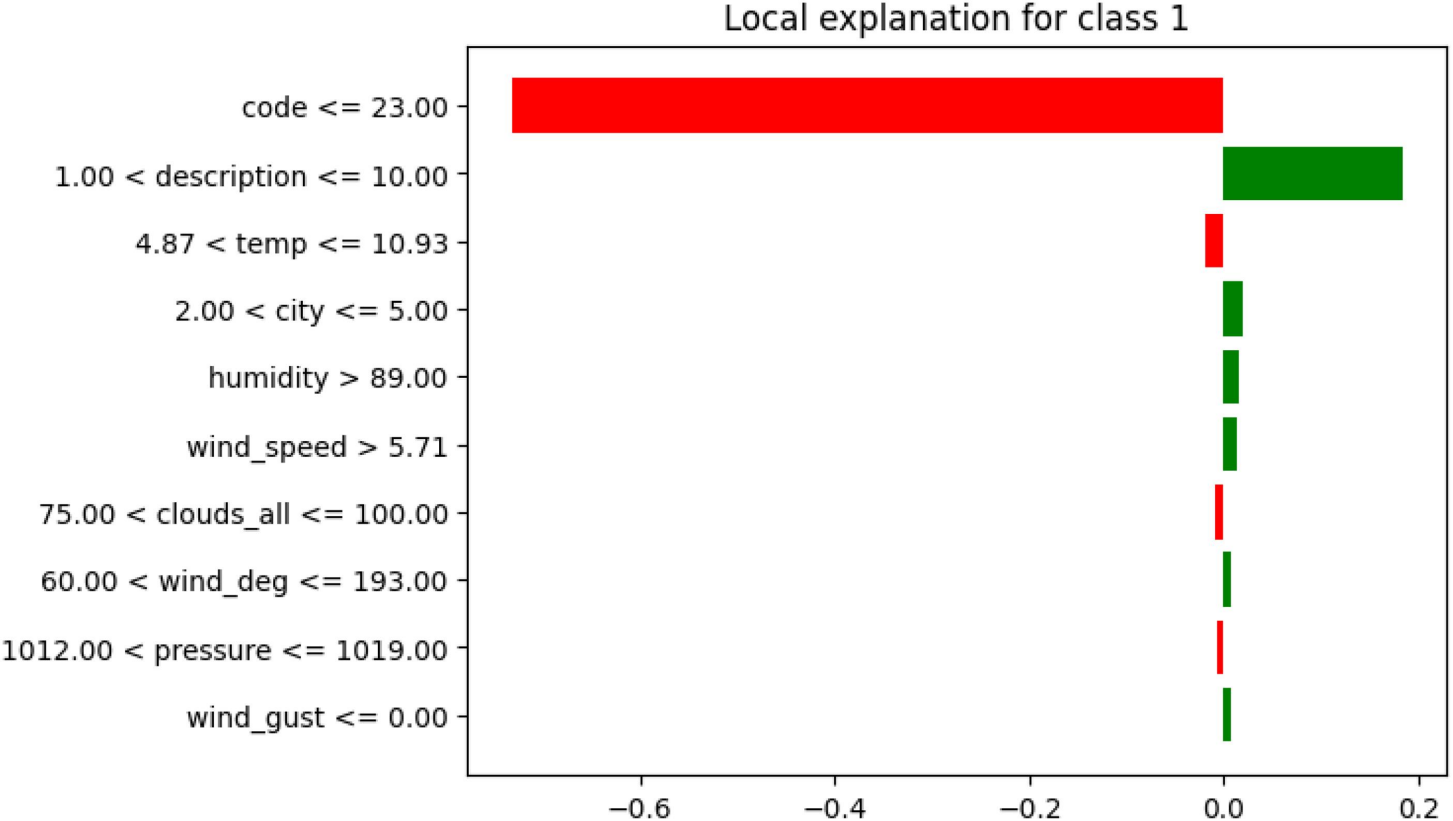
Local explanation generated by the LIME model, visualizing the contribution of each weather feature to the predicted classification outcome, providing insights into model behavior.

LIME employs different color codes to represent the value of attributes. These color codes can be used to represent the importance or significance of a component to the LIME explanation, this will allow the results to be understood. We employed LIME to generate a random forest, the associated procedure is described in Algorithm 7. Algorithm 7 has been used to explain the Decision tree classification results using LIME with ten features. The column in Figure 12 uses two color codes, such as red and green. The green color shows a positive association of the features to classify the respective class, and the red color signifies the opposite. Moreover, the diagram shows a value range for each feature toward the activity type (indoor or outdoor) classification. The visualization helps to understand which attributes contribute the most, and how much based on the probability value. According to Figure 12, the green features increase the likelihood that the current data sample is predicted to be class 1. The features of red are the opposite.

**Figure 13:**
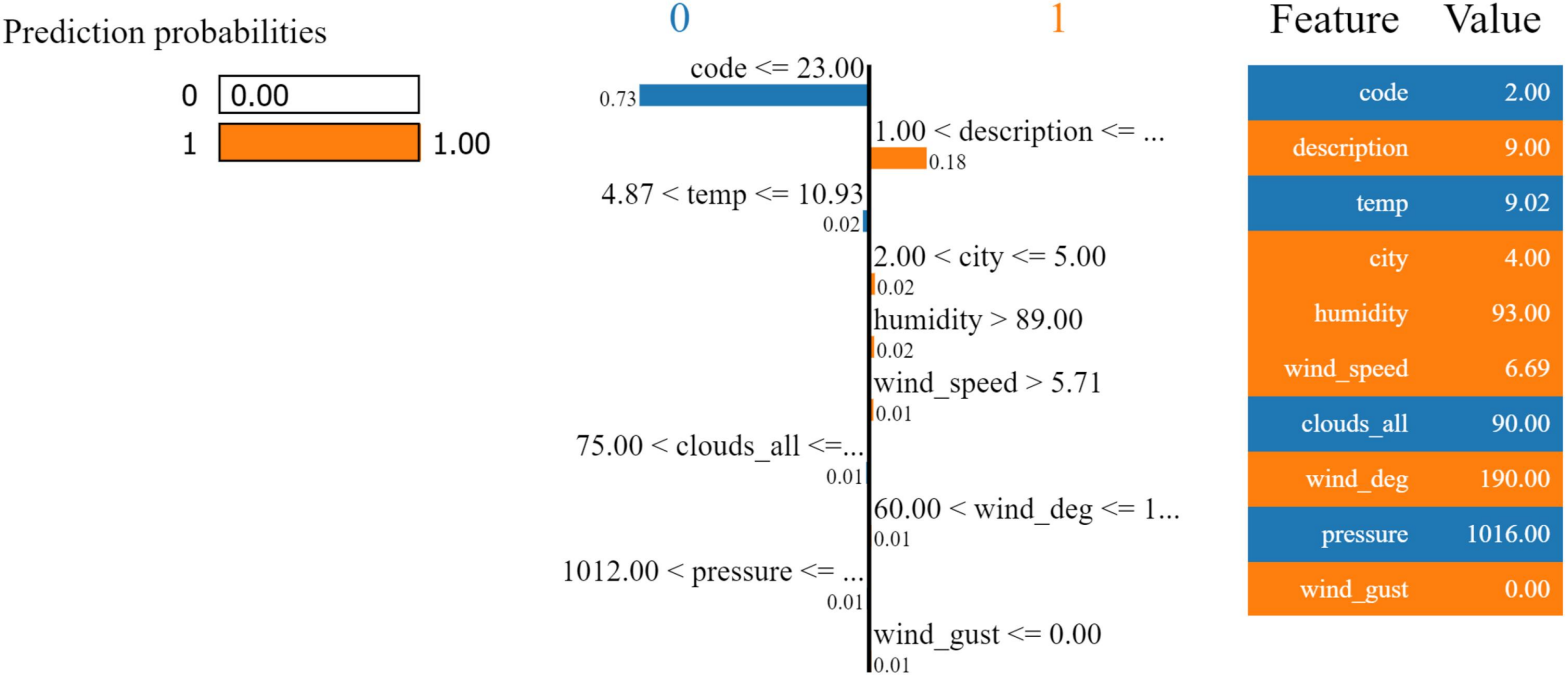
Visualization of the LIME explanation, highlighting how specific weather features influence predictions for activity type (indoor or outdoor) with probability scores for interpretability.

According to the LIME explanation (see Figure 13) The classification model produced a prediction probability close to 1.0 for the considered indoor activity class). The second column shows the features that contribute to this prediction (class 0) and those against it (class 1). It also shows the relative importance of features. The last column shows the attribute and its actual value. The probability of prediction (termed as “Prediction Probabilities” in Figure 13) in LIME provides important information about the trustworthiness and reliability of the model, which is typically between 0 and 1. Its objective is to determine the correctness or incorrectness of a forecast. Additionally, the probability of the prediction affects the significance of each feature in the generation of explanations. Those that have a significant impact on the probability of prediction are given more importance in the description, which indicates their importance in the model's decision-making process. These likelihoods are used to identify situations where the model is either very certain or unsure about its predictions. This increases the legitimacy and transparency of the classification model. In the middle diagram of Figure 13, the orange color code indicates a positive association between the features that would classify the respective class, while the light green color code indicates the opposite. The most rightward diagram is associated with Figure 12. It implies that all the identified traits have a significant impact on the activity type (indoor or outdoor) of each class.

The LIME explanations additionally align with domain knowledge regarding environmental suitability for physical activity. Features such as heavy precipitation, high humidity, strong wind speed, and poor visibility contributed positively toward indoor activity prediction because such weather conditions are commonly associated with reduced outdoor exercise participation and increased safety concerns. Conversely, mild temperature, clear sky conditions, lower humidity, and moderate wind conditions contributed positively toward outdoor activity prediction, as these environmental conditions are generally favorable for walking, cycling, and recreational outdoor activities.

#### 4.2.3 Ontology verification and recommendation generation

In Supplementary Material S5, we have included the designed and developed semantic Ontology structure in the “TTL” format for better readability. We generated instant personalized recommendations for activity types as a part of Ontology verification based on the contextual weather information and for the same, we recruited 16 adult participants (age: 18–64; Male: 12; Female: 4) with the demographic attributes as mentioned in Table 6.

**Table 6:** Participants’ demographic characteristics (*N* = 16).

Factors	Mean (*µ*)	SD (*σ)*	Min	Max	*P*25	*P*50	*P*75
Age	35.375	±7.03	21	51	30.8	35.5	39.0
Height (cm)	173.5	±8.02	158.5	184.0	167.6	173.3	180.5
Weight (Kg.)	77.0	±16.36	55.0	107.0	65.0	72.0	90.5
BMI	25.38	±3.93	19.41	31.604	22.0	25.8	27.9
Duration (days)	33.6875	±5.41	30	48	30.6	31.0	34.3
Total sedentary seconds	2449171	±1051610.5	590028	4261190	-	-	-
Total VPA seconds	41887.81	±60688.5	112	256896	-	-	-
Total MPA seconds	53231.75	±17965	23402	95730	-	-	-
Total LPA seconds	154647.1	±66540.6	32272	254332	-	-	-
Total steps	366703.3	±87202.25	52551	588132	-	-	-

However, to be precise, in Table 7 we captured potential recommendation messages of two participants as a part of the verification of personalized recommendation generation based on contextual data as example−one achieved the weekly goal, and the other did not. The same table can be elaborated with other participants’ recommendation data. The same concept can be applied to other real participants on a large scale with real-time weather data as a part of the automatic coaching and recommendation generation process. We used a trained Decision Tree classification model for activity type classification on the considered test data. We executed semantic rules and used the Jena ARQ engine to run associated SPARQL queries on the used test data. Query results have been combined to create personalized recommendations. Moreover, the test results of the eCoach system testing as formulated in the Supplementary Material S3, have also been captured in the Supplementary Material S3 for verifying the performance of the system with real-time weather data.

**Table 7:** Potential recommendations to the considered six participants based on their performances from the recommendation table.

Participant	Status	Contextual data and predicted class	Instant Recommendation message	Weekly Recommendation Message
P-1	Weekly goal achieved	Time=“9:00:56 AM”, city=“Kristiansand”, code=500, description=“light rain”, tem*p* = 6.8, pressure=1005, humidity=93, visibility=6000, wind speed=5.14, wind deg=110, wind gust=0, clouds all=75. Predicted activity type=0 (indoor activity) with 100% ac­curacy.	M-8, M-20	M-21
P-2	Weekly goal not-achieved	Time=“4:00:56 PM”, city=“Kristiansand”, code=500, description=“light rain”, tem*p* = 9.06, pressure=1010, humidity=87, visibility=10000, wind speed=5.9, wind deg=150, wind gust=7, clouds all=100. Predicted activity type=1 (outdoor activity) with 100% accuracy.	M-6, M-19	M-22

The purpose of eCoaching has been to motivate participants (through motivational recommendations) for activity on the day-(*n* + 1) based on activity progress on day-n, so that they can achieve their weekly activity goals (generic as defined by WHO or individualized) and stay more organized to maintain a healthy lifestyle. we added the example SPARQL queries for rule-based recommendation generation. We employed SPARQL queries to retrieve weather information and recommended specific physical activities based on the weather conditions, locations, and time periods in question (e.g., find a recommended physical activity for a specific location and weather condition, find a recommended physical activity for a specific weather condition, find a recommended physical activity for a specific temperature range, find weather condition for a specific location at a certain time, etc.). We utilized the OWL_MEM_MICRO_RULE_INF specification (OWL-full) to investigate the ontology structure in Jena in the TTL format and approximated the reading time to 1.0–1.2 s Moreover, we used In-memory storage, optimized rule-based reasoner OWL rules, and the Jena framework to query the ontology class, ontology, predicate, subject, and object of each sentence in *<*1.0 s, *<*1.5 s, and *<*1.5 s, respectively. The reasoning time of the Ontology model has been captured in Table 8. The HermiT reasoner performed the best without any inconsistencies.

**Table 8:** Performance comparison of different ontology reasoners available in protégé.

Reasoner(s)	Average reasoning time (sec.)
HermiT	1.0–1.5 s
Pellet	1.8–2.5 s
Fact++	1.7–2.6 s
RacerPro	2.0–2.5 s
KAON2	2.1–2.6 s

#### 4.2.4 Cross-validation stability and uncertainty analysis

To evaluate model robustness and uncertainty, we employed stratified five-fold cross-validation and recorded the mean and standard deviation of the classification metrics across all folds. Lower variance indicated stable model behavior and reduced prediction uncertainty. The Decision Tree classifier demonstrated the highest stability with a mean accuracy of 99.1% and a standard deviation of ±0.3, confirming strong generalization with minimal fluctuation across folds. The corresponding mean F1-score and MCC were 99.1% and 97.0%, respectively. In comparison, the SVC model showed higher variability with a standard deviation of ±1.2. Furthermore, the LIME explanation framework produced consistent prediction probabilities for indoor and outdoor activity classes, with most correctly classified samples showing confidence scores greater than 0.94, indicating reliable and stable classification performance (see Table 9).

**Table 9:** Five-fold cross-validation results of the decision tree classifier.

Fold	Accuracy (%)	F1-score (%)
1	98.9	98.9
2	99.0	99.0
3	99.3	99.3
4	99.1	99.1
5	99.2	99.2

#### 4.2.5 Bias, overfitting, and underfitting control strategies

Several strategies were adopted to minimize bias, overfitting, and underfitting during model development. First, stratified five-fold cross-validation was used to preserve class distribution and improve generalization. Second, highly correlated features were removed using correlation threshold analysis (≥0.8) to reduce multicollinearity. Third, ANOVA statistical testing was performed to retain only statistically significant features (*p* *<* 0*.*05). Fourth, scalability testing and training-testing validation curves were analyzed to detect overfitting tendencies.

#### 4.2.6 Computational complexity and practical feasibility

The computational complexity of the proposed classification and recommendation pipeline has been analyzed to assess its feasibility for real-time deployment. The decision tree classifier exhibits polynomial-time complexity during training and logarithmic complexity during inference, while ontology reasoning and SPARQL query execution remain within sub-second latency, supporting real-time eCoaching scenarios.

#### 4.27 Ontology scalability and query performance

To assess ontology scalability, we evaluated SPARQL query execution performance under increasing ontology instance sizes and query loads (see Table 10). The ontology has been deployed using Apache Jena Fuseki and evaluated using in-memory storage and OWL rule-based reasoning.

**Table 10:** Ontology scalability and SPARQL query execution performance using apache Jena fuseki with OWL rule-based reasoning.

Ontology Size	No. of Queries	Average Query Time (sec.)	Reasoning Time (sec.)	Status
100 instances	10	0.08	0.9	Stable
200 instances	25	0.15	1.1	Stable
300 instances	50	0.24	1.3	Stable
400 instances	75	0.41	1.6	Stable
500 instances	100	0.73	2.1	Stable

## 5 Discussion

It is a five-fold study. First, real contextual data and participant data had been collected. Second, based on contextual data, we classified the dataset into activity types (indoor: 1 and outdoor: 0) with a traditional machine learning algorithm. Third, we designed and developed an Ontology for semantic annotation and reasoning. Fourth, we used annotated predictive analysis and participant data for recommendation generation using SPARQL query language on designed and developed Ontology. Fifth, testing of physical activity eCoach recommendation system based on real weather data.

### 5.1 Model selection for predictive analysis

Deep learning and data augmentation were not used in this study due to the nature of the problem, the size of the dataset, and the interpretability requirements of the eCoach system. Deep learning models, while powerful, typically require massive amounts of labeled data to train effectively, and the available weather dataset, while substantial, did not demonstrate the scale or complexity to justify the use of deep architectures. Furthermore, deep learning models often act as black boxes, making it difficult to explain their predictions—a critical limitation for a system that aims to provide clear and interpretable recommendations. Instead, simpler machine learning algorithms such as decision trees were favored due to their ability to efficiently handle moderately sized datasets and provide clear explanations through visualization and feature importance analyses. The Decision Tree classification is an intuitive and interpretable algorithm that efficiently handles both categorical and numerical data, which is necessary for processing a variety of weather attributes. The classifier achieved better performance compared to the alternatives, with an accuracy of 99.1% and equally high scores for precision, recall, and MCC. The transparent decision-making process enables easy visualization of decision rules, which meets the eCoach system's need for explainability. The use of entropy as a splitting criterion optimizes its performance, providing highly homogeneous subsets, making the model robust and accurate. Compared to other classifiers such as logistic regression and support vector machines, the low computational burden and ability to provide interpretable results make Decision Tree the most effective choice for this application. Data augmentation, which is commonly used to artificially expand data sets by creating variants of existing samples, was not applicable here because the collected weather data inherently captured sufficient variability across time and location. Given the need for logical reasoning, semantic annotation, and transparent decision-making, a hybrid approach combining machine learning and semantic ontology proved more suitable for generating personalized and contextually sensitive activity recommendations.

### 5.2 Personalized recommendation generation

Average execution times for SPARQL queries were recorded between 0.1 s and 0.3 s (sec). The semantic rules (or propositional logic) used in this study represent the generation logic of personalized recommendation information, and rule-based binary reasoning (if → 1, else → 0) helps explain the formation of personal activity type recommendation information. Due to the false positive situation, it is crucial to adopt a fully data-driven approach to generate personalized healthcare recommendations. Therefore, predictive modeling and annotated rule sets can add value to personalized health recommendations. Personalization is essential for adaptive health coach recommendation systems to enhance the user's experience, privacy, and control over their data and preferences. We used a tuple database as a knowledge base to store customizable recommendation messages and rules. A tuple database uses tuples as the fundamental unit of data storage and retrieval. Both the asserted and inferred information attained from the reasoning method had been effective in determining the most appropriate recommendation message. To verify the instant personalized recommendation generation in dummy participants' data, we used the best-performing classifier model for their activity type classification and semantic Ontology for annotation, reasoning, and querying. We used an incremental learning approach to handle the growing weather dataset. The incremental learning approach has helped in activity type classification on the day-(*n* + 1) based on model training with contextual data up to day-n. Moreover, we have used three standard messages in recommendation visualization to motivate participants based on their weekly goal accomplishment (good work, satisfactory performance, and improved performance).

**Figure 14:**
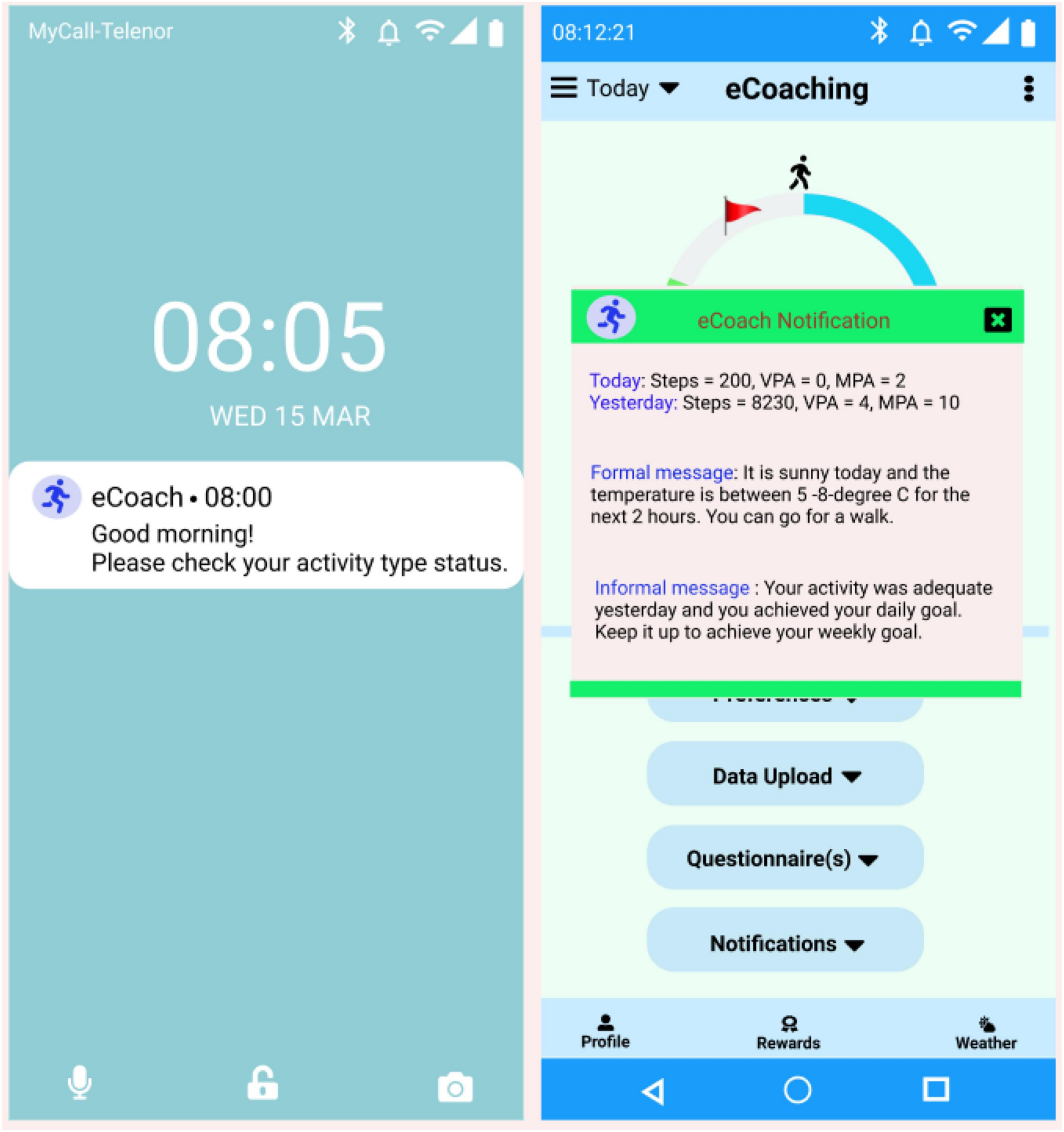
Example of an instant recommendation delivered as a pop-up notification in the eCoach system, demonstrating contextual and personalized suggestions based on weather conditions and activity goals.

We conceptualized that the recommendations can be of three types based on the user's choice and time for delivery−instant recommendation pop-up alert based on the sudden weather change forecast data (e.g., Now the weather is sunny, and it will be for next three hours. You can go for a walk/running/cycling/other outdoor activities and don't forget to wear your activity tracker), daily recommendation (e.g., Today, there will be heavy snowfall today. Therefore, plan your indoor activities accordingly), and weekly recommendation (e.g., Last week you were 30 min less active. This week, the weather will be sunny and cloudy. Please plan your activities accordingly). The frequency of recommendation generation and delivery is user preference-based. An example of instant pop-up recommendation generation and its visualization has been captured in Figure 14.

By testing the physical activity recommendation system with real weather data under various scenarios (see Supplementary Material S3), it ensures that the system can handle different weather conditions and provide relevant and practical activity suggestions to users in real-world situations. Our proposed eCoach recommendation system gives design flexibility due to the modular design pattern and produces accurate context-based recommendations. Overall, this study rigorously focuses on automating the personalized activity type recommendation generation with machine learning, personal preference information, adjustable rule base, and their integration with a semantic network for reasoning and meaningful querying for personalized and contextual recommendations. The dataset we used were medium, and we thought they might be biased. High bias can lead to model under-fitting. Therefore, we used the MCC metric to understand the ML classification model's performance better. Moreover, the use of machine learning algorithms in combination with semantic Ontology has been evident to generate contextual, evidence-based, goal-based, personalized, and hybrid recommendation generation in an eCoach system. An environment for the proposed algorithm implementation has been simulated in Python with limited dummy data, and made available in HTML format as Supplementary Material S6. This study is intentionally positioned as a technical proof of concept rather than a full-scale system validation study. Its primary goal is to demonstrate the technical feasibility of integrating machine learning, semantic ontology, and rule-based reasoning to generate personalized and contextual activity recommendations from outdoor weather data. Using real contextual and dummy participant data was a deliberate choice to rigorously evaluate the system's algorithmic design, classification accuracy, semantic reasoning framework, and recommendation generation logic before scaling to real-world deployment. This approach allows for careful testing and optimization of the underlying models without the confounding factors that often introduce uncontrolled real-world data collection. Although the present study primarily focuses on the technical validation of contextual recommendation generation, usability evaluation remains an important aspect for future deployment of eCoaching systems. Future work will incorporate user-centered usability studies involving constructs such as perceived usefulness, ease of use, recommendation satisfaction, user engagement, adherence behavior, and trust in explainable recommendations.

### 5.3 Integration of machine learning, personal preferences, and semantic ontology in eCoaching

This study extends our previous ontology-based and predictive modeling frameworks for activity recommendation (see Table 1) by incorporating contextual weather-driven classification and explainable AI mechanisms for transparent recommendation generation. The integration of ML, personal preferences, and semantic ontology significantly increases the performance of the eCoach system, enabling personalized, meaningful, and contextual activity recommendations. Machine learning provides the ability to analyze contextual weather data and predict whether indoor or outdoor activities are suitable with high accuracy. For example, a Decision Tree classifier is used to classify activity types based on weather characteristics, and its integration with explainable AI techniques such as LIME provides transparency in predictions, thereby building user trust. The Decision Tree classifier with entropy produced the best output in terms of accuracy and execution time, as it effectively handled the categorical and numerical data. The Decision Tree algorithm with Entropy worked by recursively partitioning a dataset based on the features that provided the highest information gain, calculated as the difference between the entropy of the parent node and the weighted average entropy of the child nodes. By partitioning data in this way, the Decision Tree can efficiently create a set of rules that can accurately classify new examples. The statistical analysis showed that in Southern Norway, due to the favorable weather condition, the probability of doing outdoor activity is more than indoor activities. Personal preferences further refine these recommendations by taking into account individual choices such as preferred activity types, times, or notification formats. This customization ensures that recommendations are relevant and delivered in a way that is consistent with user behavior, increasing user engagement and compliance with the advice provided. The semantic ontology acts as an organizing framework, systematically representing knowledge by integrating weather data, activity types, and user-specific rules. Through logical reasoning and structured queries, the semantic ontology ensures that recommendations remain consistent and adapt to complex scenarios. The use of SPARQL queries enables efficient retrieval of actionable insights from the ontology, providing rapid response and scalability. Together, these components enable the eCoach system to dynamically adapt to changes in the environment and user needs, making its recommendations effective and personalized.

The semantic ontology and the explainable AI module serve complementary but distinct purposes within the proposed framework. The ontology is responsible for semantic knowledge representation, logical reasoning, rule execution, and contextual recommendation retrieval through SPARQL querying. In contrast, the explainable AI module, namely LIME, is used to interpret and explain the predictions produced by the machine learning classifier. Despite the advantages of the proposed framework, several practical challenges remain. First, ontology maintenance and rule management may become increasingly complex as the number of contextual variables and recommendation rules grows. Second, dependence on external weather APIs introduces potential risks related to data latency, incomplete observations, and service availability. Third, highly interpretable models such as Decision Trees may sacrifice predictive flexibility compared to more complex deep learning approaches. Finally, user adaptation and long-term engagement with recommendation systems remain challenging in real-world deployment scenarios.

### 5.4 Role of external weather data in context-based recommendations

Weather data plays a key role in providing context-based recommendations, as outdoor conditions directly impact the feasibility and safety of outdoor activities. Adverse weather conditions, such as heavy rain, extreme heat, or snow, can discourage outdoor activities and require indoor alternatives. By incorporating real-time weather data, eCoach ensures that recommendations are based on environmental realities, making them practical and reducing risk to users. This data is integrated into the recommendation model via APIs such as OpenWeatherMap, which provides information on features such as temperature, humidity, precipitation, and wind speed. These features are processed by an ML classifier to determine the suitability of indoor or outdoor activities. Additionally, semantic rules in the ontology map specific weather conditions to their corresponding activity types. This integration ensures that the system generates precise recommendations that are consistent with both current and forecasted weather conditions, increasing user compliance and promoting year-round activity engagement.

### 5.5 Role of semantic ontology and SPARQL in recommendation generation

Semantic ontology plays a key role in ensuring logical consistency and adaptability of recommendations. By providing a formal, hierarchical structure to represent relationships between weather conditions, activity types, and user preferences, the ontology enables eCoach to reason about structured data. Logical axioms embedded in the ontology ensure that recommendations follow consistent rules, even in complex scenarios involving overlapping or conflicting conditions. The ontology bridges the gap between raw weather data and actionable insights, supporting both machine reasoning and human understanding. The SPARQL query engine further enhances this system by enabling efficient ontology searching. For example, queries such as “get relevant actions for a given temperature range” or “find recommendations for forecasted rain” enable dynamic generation of context-based recommendations. SPARQL ensures that recommendations are delivered efficiently and logically, as predicted by the ML model. This combination of semantic reasoning and structured searching provides a solid framework for generating personalized, relevant, and logically consistent recommendations, increasing the overall user experience and trust in the eCoach system. Supplementary Material S7 lists used SPARQL queries to validate ontology structure, retrieve contextual weather concepts, identify activity-type classes, verify recommendation-generation relations, and support the test cases.

### 5.6 Validation with simulated participant data

The use of simulated participant data in this study served as a technical validation tool to test the feasibility and functionality of the proposed recommendation framework before implementing it in real-world scenarios. This enabled the evaluation of the classification algorithm, semantic ontology, and SPARQL query processing to generate personalized recommendations based on contextual weather conditions. The dummy data facilitated incremental learning experiments, ensuring that the system could dynamically dummy to growing data sets and provide consistent recommendations over time. Furthermore, it enabled the simulation of various test cases to assess the adaptability, logical consistency, and rule-based reasoning of the system under different weather scenarios and activity goals. While this approach focused on demonstrating a technical proof of concept, future research will include real-world user data to validate the practical applicability of the system and assess its impact in real-world conditions. While effective in testing the technical feasibility of algorithms, dummy data lack the dynamic patterns, behavioral nuances, and unpredictability observed in real users. This limitation can lead to oversimplified scenarios in which a system performs exceptionally well in controlled tests but may encounter challenges in adapting to different preferences, changing routines, and unexpected interactions in real-world contexts. Furthermore, dummy data cannot model the psychological and motivational factors that influence physical activity decisions, which are essential for assessing the practical effectiveness of recommendations. Despite these challenges, using dummy data was a deliberate choice in this proof-of-concept study to focus on validating the algorithmic framework, classification accuracy, and semantic reasoning capabilities before scaling to real-world implementation. This allowed us to rigorously evaluate the system architecture, machine learning pipelines, and recommendation logic under controlled conditions, providing a solid foundation for further development. However, the use of simulated participant data is not critical for the present study because the simulated participant profiles are designed to emulate real time system behavior. They are streamed and updated in a time dependent manner to mirror how actual user signals and preferences would arrive and evolve in deployment. Since the core objective here is to validate real time weather driven classification, semantic annotation, and rule based recommendation generation, the simulated data still has provided a realistic operational setting for testing system responsiveness and logical consistency. Therefore, the absence of real participants does not substantially weaken the technical conclusions, but rather postpones the user level validation to the next stage of research.

### 5.7 Limitations and future scope

In realistic coaching for weekly or monthly goals, as part of continuous monitoring, the eCoach module generates timely personalized and contextual recommendations based on daily activity results, followed by predictive analysis for achieving weekly goals. Furthermore, it is not actual coaching, but conceptual modeling using machine learning and semantic techniques. The process is highly personalized and therefore does not include the concept of group similarity in recommendation generation. In the future, we will extend this study with a group-based meta-heuristic approach, combining the idea of collaborative filtering. We will further analyze the usefulness of density-based spatial clustering, sessions, and criteria in our future group-based lifestyle recommendation generation. Overall, this outdoor weather-based eCoach physical activity recommendation system can help users achieve their fitness goals, stay motivated, and positively impact their overall health and well-being. Furthermore, the used dataset primarily reflects Nordic climate conditions and therefore may not fully generalize to tropical, desert, or continental climates. Future work will therefore focus on geographically diverse real-world datasets and longitudinal deployment studies. This study highlights several promising directions for future research in eCoaching systems. *First*, the integration of real-time feedback mechanisms, particularly in mobile eCoaching platforms, presents both challenges and opportunities that require further exploration. Additionally, empirical testing with real users would help assess user engagement, satisfaction, and long-term behavioral changes, offering deeper insights into the practical usability and scalability of the system. Future iterations of the system will integrate incremental learning methods to handle growing data sets and adaptive feedback mechanisms to further improve recommendation precision and user satisfaction. This phased approach ensures that the technical foundation established in this study can be seamlessly extended to a real-world application, making the transition from proof-of-concept to deployment both efficient and evidence-based. *Second*, ensuring the fairness of user recommendations remains critical; developing algorithms that provide equal support to different user groups is essential. *Third*, addressing ethical issues such as user privacy and autonomy is necessary as eCoaching systems become increasingly common in promoting healthier lifestyles. *Fourth*, a key area is the integration of real-time feedback mechanisms, which have been shown to increase user engagement and learning outcomes. *Fifth*, conducting longitudinal evaluation studies is essential to assess the sustained effectiveness of eCoaching interventions and to rigorously evaluate the system's resilience, adaptability, and capacity under diverse real-world scenarios. Such longitudinal studies can provide insights into how these systems influence user behavior and learning over longer periods. The goal of ongoing and future work is to bridge the gap between algorithmic validation and practical implementation, ultimately leading to the creation of a comprehensive, evidence-based framework for improving physical activity and contextual health outcomes.

## 6 Conclusion

This study presented a novel approach to designing and developing an intelligent eCoach system by integrating standard machine learning technology, personal preferences, and a semantic ontology. The proposed system generates automatic, meaningful, contextual, and personalized activity-type recommendations to help individuals achieve their personal activity goals. Our approach relies on external weather data to provide context-based recommendations by predicting suitable activity types (indoor or outdoor) based on current and forecast weather conditions. The study mainly focused on collecting real external weather data for prediction modeling and utilized dummy participant data (activity and preference) to validate the proposed recommendation generation algorithm for different possible cases. It is important to note that this study is conceptual in nature, and its real-time evaluation with actual participant data remains a future research direction. Semantic ontology played a crucial role in semantic annotation, knowledge representation, reasoning, and rule-based recommendation generation using the SPARQL query engine. The Decision Tree classifier outperformed other classifiers in this binary classification problem, while LIME proved useful in explaining the classification results through probability distribution and feature importance. Additionally, the ontology verification module functioned as a rule-based decision support system, ensuring the generation and delivery of personalized recommendation messages followed a logically consistent structure. In conclusion, this study has demonstrated the potential of integrating machine learning, personal preferences, and semantic ontology in the design and development of an eCoach system for generating personalized activity-type recommendations. The proposed system has practical implications for promoting healthier lifestyles and could be further refined and evaluated with actual participant data in future research. Moreover, by utilizing a diverse set of scenarios, we have successfully assessed the physical activity eCoach recommendations system's resilience, adaptability, and capacity to provide relevant activity suggestions in various weather conditions and user preferences. It facilitates the verification that the system is capable of handling different real-world situations and providing valuable advice to users in a variety of scenarios.

## Data Availability

The datasets presented in this study can be found in online repositories. The names of the repository/repositories and accession number(s) can be found in the article/Supplementary Material.
