# Supplementary material for: Contextual recommendation modeling in eCoaching with machine learning, X-AI, and semantic ontology
Source: Front Digit Health. 2026 Jul 15;8:1811976. doi: 10.3389/fdgth.2026.1811976 (PMC13416675; doi:10.3389/fdgth.2026.1811976)
Supplement: Supplementary file 1 [file Datasheet1.pdf]

Table S-1: The eCoaching approaches.

Approach	Description
Goal-oriented approach	This approach involves focusing on a specific goal that the individual wants to achieve. The eCoaching system will work with individuals to set clear, measurable, and attainable goals and develop a plan to achieve them.
Action-oriented approach	This approach focuses on taking actions to achieve desired results. The eCoaching system will encourage individuals to take steps towards their goals e.g., break down goals into smaller, achievable steps and track progress.
Feedback-oriented approach	This approach involves providing feedback on an individual's progress and performance. The eCoaching system monitors and evaluates an individual's behavior and provides feedback on what is working well and where improvement can be made.
Self-reflection approach	This approach encourages individuals to reflect on their actions, thoughts and emotions. eCoaching systems can use reflective exercises, such as journaling or meditation, to help individuals gain insight into their own behavior and thought patterns.
Motivational approach	This approach is about motivating and motivating individuals to achieve their goals. eCoaching systems can use techniques such as positive reinforcement, inspiration, and goal visualization to help individuals stay motivated and committed to their goals.
Evidence-based approach	This approach involves using the best available scientific evidence to make a decision. Health coaches can use clinical guidelines, research, and other sources of evidence to make recommendations for their clients.
Patient-centered approach	This approach places the patient at the center of the decision-making process. Health coaches work with their clients to understand their preferences, values, and goals, and develop a program that fits their unique needs.
Behavioral approach	This approach focuses on changing behaviors that lead to adverse health outcomes. Wellness coaches can use techniques like goal setting, self-monitoring, and positive reinforcement to help clients develop healthier habits.
Collaborative approach	This approach involves working with the client as a partner in decision making. Health coaches engage in open dialogue and shared decision-making with their clients to develop a mutually agreed and achievable plan.
Strengths-based approach	This approach focuses on identifying and building on the strengths and resources the client already has. A health coach can help clients identify their existing skills and use them to achieve their health goals.
