# Supplementary material for: Contextual recommendation modeling in eCoaching with machine learning, X-AI, and semantic ontology
Source: Front Digit Health. 2026 Jul 15;8:1811976. doi: 10.3389/fdgth.2026.1811976 (PMC13416675; doi:10.3389/fdgth.2026.1811976)
Supplement: Supplementary file 2 [file Datasheet2.pdf]

Table S-2: The recommendation table with potential recommendation messages.

Message Id	Message(s)
M-1	"Good morning! It's a beautiful day outside with sunny skies and moderate temperatures. How about going for a hike or a jog in the park?"
M-2	"Hello! It's quite humid outside today, so make sure to stay hydrated if you're planning on any outdoor activities. Consider taking a swim or doing an indoor workout instead."
M-3	"Hello! It looks like it's going to be a bit cold today. Be sure to wear layers if you're going to be outside. How about a brisk walk or an indoor game?"
M-4	"Hello! The weather is perfect for outdoor activities today with clear skies and a gentle breeze. Consider taking a bike ride or playing an outdoor activity."
M-5	"Good afternoon! The temperature is increasing today; therefore, be careful if you're planning on any strenuous outdoor activities. How about a yoga class or a gentle swim?"
M-6	"Hello! It's going to be cloudy and overcast today, hence, take advantage of the cooler weather by going for a run or a walk."
M-7	"Good morning! It's a bit windy today, so be cautious if you're planning on any outdoor activities that involve balance or coordination. Consider a low-impact workout like stretching."
M-8	"Hello! It's raining outside today. Consider an indoor cycling class or a dance or swimming or yoga workout at home."
M-9	"Hello! The weather is perfect for team sports today, so gather some friends for a game of soccer or basketball or similar."
M-10	"Hello! It's quite cold outside today, so make sure to bundle up if you're planning on any outdoor activities. Consider a winter sport like skiing or snowshoeing."
M-11	"Good afternoon! It's going to be very hot and humid today, so be sure to take extra precautions if you're planning on exercising outside. Consider an early morning or evening workout instead."
M-12	"Hello! It's going to be stormy today, so avoid any outdoor activities that could be dangerous. Consider a workout at home or a yoga class."
M-13	"Good morning! The weather is perfect for water sports today, so head to the beach or pool for a swim or a similar session."
M-14	"Hello! The air quality is poor today, so consider an indoor workout if you're sensitive to pollution. How about a dance class or a weightlifting session?"
M-15	"Hello! It's going to be very windy today, so be careful if you're planning on any outdoor activities that involve high speeds. Consider a low-impact workout like tai chi or a meditation class."
M-16	"Hello! It's going to be very hot and dry today, so make sure to stay hydrated and avoid exercising during the hottest parts of the day. How about a morning or evening workout?"
M-17	"Good afternoon! The weather is perfect for a game of tennis or a round of golf today!"
M-18	"Hello! It's going to be quite cold and windy today, so make sure to dress appropriately if you're planning on any outdoor activities. Consider an indoor workout like a spin class or a similar session."
M-19	"Good morning! The weather is perfect for a hike, or a nature walk today, so grab a friend and enjoy the fresh air and scenery."
M-20	"Hello! The weather is not ideal for outdoor activities today with heavy rain and strong winds. Consider a workout at home or a meditation session to relax and de-stress."
M-21	“Well Done! Keep it up. You have achieved the weekly activity goal to stay moderate physically active.”
M-22	“You need to work hard as you have not achieved the weekly activity goal to stay moderate physically active.”
