# Supplementary material for: Contextual recommendation modeling in eCoaching with machine learning, X-AI, and semantic ontology
Source: Front Digit Health. 2026 Jul 15;8:1811976. doi: 10.3389/fdgth.2026.1811976 (PMC13416675; doi:10.3389/fdgth.2026.1811976)
Supplement: Supplementary file 3 [file Datasheet3.pdf]

Table S-3: The system test cases and status.

Test Id	Weather conditions	Action (Input and Output)	Status
1	Sunny Day	Input: Weather forecast indicates a sunny day with clear skies and pleasant temperature.<br><br>Expected Output: Recommend outdoor activities.	Passed
2	Rainy Day	Input: Weather forecast indicates rain and cloudy weather.<br><br>Expected Output: Recommend indoor activities.	Passed
3	Hot Day	Input: Weather forecast indicates a hot day with high temperatures.<br><br>Expected Output: Recommend water-based activities like swimming or going to a water park, or indoor activities in air-conditioned spaces.	Passed
4	Windy Day	Input: Weather forecast indicates strong winds.<br><br>Expected Output: Recommend activities that are less affected by wind, such as indoor sports or visiting a wind-sheltered location.	Passed
5	Snowy Day	Input: Weather forecast indicates snowfall.<br><br>Expected Output: Recommend winter activities like skiing, snowboarding, or building a snowman.	Passed
6	Mixed Weather Conditions	Input: Weather forecast indicates a mix of sunny and rainy periods throughout the day.<br><br>Expected Output: The recommendation system should offer a diverse range of activity suggestions that accommodate the changing weather conditions.	Passed
7	Extreme Weather	Input: Weather forecast indicates extreme weather conditions like storms, or heatwaves.<br><br>Expected Output: The recommendation system should prioritize user safety and avoid suggesting outdoor activities that might be dangerous during extreme weather.	Passed
8	Real-time Weather Updates	The recommendation system should adapt and provide updated activity suggestions based on the latest weather information	Passed
9	Unavailable Weather Data	Input: Weather data for the current location is unavailable or returns an error.<br><br>Expected Output: The recommendation system should handle the absence of weather data gracefully and provide alternative suggestions based on default or historical weather patterns for that location.	Passed
10	Incorrect Weather Data Format	Input: Weather data received from the weather API is in an unexpected or incorrect format (e.g., XML instead of JSON)<br><br>Expected Output: The recommendation system should handle the parsing error and notify the user about the data issue without crashing.	Passed

11	Zero Weather Data	Input: The weather data returned has missing values or zeros for temperature and other weather parameters.<br><br>Expected Output: The recommendation system should handle missing or zero values and provide a reasonable default recommendation, considering the data limitations.	Passed
12	Invalid User Location	Input: The user's location provided is invalid or not recognized by the weather API.<br><br>Expected Output: The recommendation system should handle the invalid location and prompt the user to provide a valid location.	Passed
13	High Humidity	Input: Weather forecast indicates high humidity levels.<br><br>Expected Output: Recommend activities suitable for humid conditions, such as swimming, staying indoors with air conditioning, or engaging in water-based sports.	Passed
14	Foggy Day	Input: Weather forecast indicates foggy conditions.<br><br>Expected Output: Recommend activities that are safe and visible in foggy weather, such as indoor activities or walking in well-lit areas.	Passed
15	User Allergies or Health Conditions	Input: The user has allergies or health conditions that restrict certain outdoor activities, regardless of weather conditions.<br><br>Expected Output: The recommendation system should consider the user's health restrictions and avoid suggesting activities that might trigger allergies or worsen health conditions.	Passed
16	Sunny and Warm	Input: Real weather data indicates a sunny day with warm temperatures.<br><br>Expected Output: Recommend outdoor activities.	Passed
17	Rainy and Cold	Input: Real weather data indicates rainy and cold weather.<br><br>Expected Output: Recommend indoor activities.	Passed
18	Hot and Humid	Input: Real weather data indicates hot and humid conditions.<br><br>Expected Output: Recommend water-based activities like swimming or going to a water park or suggest indoor activities in air-conditioned spaces.	Passed
19	Low Light Conditions	Input: Real weather data indicates low light conditions due to overcast skies or early morning/evening hours.<br><br>Expected Output: Recommend activities that are suitable for low-light conditions or provide safety tips for outdoor activities during such times.	Passed
20	Travel Destination	Input: User is traveling to a different location with a different weather forecast.<br><br>Expected Output: The recommendation system should provide destination-specific activity suggestions based on the weather conditions at the travel destination	Passed
