# Supplementary material for: Contextual recommendation modeling in eCoaching with machine learning, X-AI, and semantic ontology
Source: Front Digit Health. 2026 Jul 15;8:1811976. doi: 10.3389/fdgth.2026.1811976 (PMC13416675; doi:10.3389/fdgth.2026.1811976)
Supplement: Supplementary file 4 [file Datasheet4.pdf]

Table S-4: The ANOVA typ = 2 test outcomes to find out the feature dependency on the predicted class "activity_type".

Feature	Sum_sq	df = degree of freedom	F-value	PR (> F)
City	5.598214	1.0	33.18808	8.373414e-09
Code	148.860671	1.0	884.721268	3.673732e-194
Description	11892.487560	1.0	89099.801493	0.0
Pressure	48.629301	1.0	288.508707	1.117985e-64
humidity	497.457083	1.0	2974.783473	0.0
visibility	195.872447	1.0	1165.089799	6.436169e-255
wind_speed	0.137170	1.0	0.813113	0.367202
wind_deg	242.697866	1.0	1444.808993	1.802033e-315
wind_gust	417.276607	1.0	2491.767112	0.0
clouds_all	2035.580997	1.0	12513.647454	0.0
