# Supplementary material for: Contextual recommendation modeling in eCoaching with machine learning, X-AI, and semantic ontology
Source: Front Digit Health. 2026 Jul 15;8:1811976. doi: 10.3389/fdgth.2026.1811976 (PMC13416675; doi:10.3389/fdgth.2026.1811976)
Supplement: Supplementary file 5 [file Datasheet5.pdf]

```
@prefix : <http://example.com/ontology#> .
@prefix owl: <http://www.w3.org/2002/07/owl#> .
@prefix rdf: <http://www.w3.org/1999/02/22-rdf-syntax-ns#> .
@prefix xml: <http://www.w3.org/XML/1998/namespace> .
@prefix xsd: <http://www.w3.org/2001/XMLSchema#> .
@prefix rdfs: <http://www.w3.org/2000/01/rdf-schema#> .
@prefix ontology: <http://example.com/ontology#> .
@base <http://example.com/ontology#> .

<http://example.com/ontology#> rdf:type owl:Ontology ;
                         owl:versionIRI <http://example.com/ontology#1.0> ;
                         rdfs:label "Example Ontology" .

#################################################################
#    Object Properties
#################################################################

###  http://example.com/ontology#hasActivityStatus
ontology:hasActivityStatus rdf:type owl:ObjectProperty ;
                   rdfs:domain ontology:RecommendationGeneration ;
                   rdfs:range ontology:ActivityStatus ;
                   rdfs:label "has activity status" .


###  http://example.com/ontology#hasActivityType
ontology:hasActivityType rdf:type owl:ObjectProperty ;
                  rdfs:domain ontology:RecommendationGeneration ;
                  rdfs:range ontology:ActivityType ;
                  rdfs:label "has activity type" .


###  http://example.com/ontology#hasCity
ontology:hasCity rdf:type owl:ObjectProperty ;
            rdfs:domain ontology:PersonalInformation ;
            rdfs:range ontology:City ;
            rdfs:label "has city" .


###  http://example.com/ontology#hasGoalStatus
ontology:hasGoalStatus rdf:type owl:ObjectProperty ;
                 rdfs:domain ontology:RecommendationGeneration ;
                 rdfs:range ontology:GoalStatus ;
                 rdfs:label "has goal status" .


###  http://example.com/ontology#hasPreferences
ontology:hasPreferences rdf:type owl:ObjectProperty ;
                 rdfs:domain ontology:PersonalInformation ;
                 rdfs:range ontology:PersonalPreferences ;
                 rdfs:label "has preferences" .


###  http://example.com/ontology#hasRecommendation
ontology:hasRecommendation rdf:type owl:ObjectProperty .
```

```
###  http://example.com/ontology#hasTimestamp
ontology:hasTimestamp rdf:type owl:ObjectProperty ;
            rdfs:domain ontology:RecommendationGeneration ;
            rdfs:range ontology:Timestamp ;
            rdfs:label "has timestamp" .


###  http://example.com/ontology#hasWeatherData
ontology:hasWeatherData rdf:type owl:ObjectProperty ;
             rdfs:domain ontology:RecommendationGeneration ;
             rdfs:range ontology:ExternalWeatherData ;
             rdfs:label "has weather data" .


#################################################################
#    Data properties
#################################################################

###  http://example.com/ontology#hasPersonalInformation
ontology:hasPersonalInformation rdf:type owl:DatatypeProperty ;
                  rdfs:domain ontology:RecommendationGeneration ;
                  rdfs:range xsd:string ;
                  rdfs:label "has personal information" .


#################################################################
#    Classes
#################################################################

###  http://example.com/ontology#Actionable
ontology:Actionable rdf:type owl:Class ;
           rdfs:subClassOf ontology:RecommendationGeneration .


###  http://example.com/ontology#ActivityStatus
ontology:ActivityStatus rdf:type owl:Class ;
             rdfs:label "Activity Status" .


###  http://example.com/ontology#ActivityType
ontology:ActivityType rdf:type owl:Class ;
            rdfs:label "Activity Type" .


###  http://example.com/ontology#Activity_Level
ontology:Activity_Level rdf:type owl:Class ;
             rdfs:subClassOf ontology:GoalStatus .


###  http://example.com/ontology#Address
ontology:Address rdf:type owl:Class ;
          rdfs:subClassOf ontology:PersonalInformation .
```

```
###  http://example.com/ontology#City
ontology:City rdf:type owl:Class ;
          rdfs:label "City" .


###  http://example.com/ontology#Cloud_All
ontology:Cloud_All rdf:type owl:Class ;
              rdfs:subClassOf ontology:ExternalWeatherData .


###  http://example.com/ontology#Country
ontology:Country rdf:type owl:Class ;
            rdfs:subClassOf ontology:City .


###  http://example.com/ontology#Date
ontology:Date rdf:type owl:Class ;
          rdfs:subClassOf ontology:Timestamp .


###  http://example.com/ontology#Description
ontology:Description rdf:type owl:Class ;
                rdfs:subClassOf ontology:ExternalWeatherData .


###  http://example.com/ontology#Email
ontology:Email rdf:type owl:Class ;
           rdfs:subClassOf ontology:PersonalInformation .


###  http://example.com/ontology#ExternalWeatherData
ontology:ExternalWeatherData rdf:type owl:Class ;
                      rdfs:label "External Weather Data" .


###  http://example.com/ontology#FirstName
ontology:FirstName rdf:type owl:Class ;
              rdfs:subClassOf ontology:PersonalInformation .


###  http://example.com/ontology#GoalStatus
ontology:GoalStatus rdf:type owl:Class ;
               rdfs:label "Goal Status" .


###  http://example.com/ontology#Ground_Level
ontology:Ground_Level rdf:type owl:Class ;
                rdfs:subClassOf ontology:ExternalWeatherData .


###  http://example.com/ontology#High
ontology:High rdf:type owl:Class ;
          rdfs:subClassOf ontology:ActivityStatus .
```

```
###  http://example.com/ontology#Humidity
ontology:Humidity rdf:type owl:Class ;
                rdfs:subClassOf ontology:ExternalWeatherData .


###  http://example.com/ontology#Indoor
ontology:Indoor rdf:type owl:Class ;
              rdfs:subClassOf ontology:ActivityType .


###  http://example.com/ontology#LastName
ontology:LastName rdf:type owl:Class ;
                rdfs:subClassOf ontology:PersonalInformation .


###  http://example.com/ontology#Latitude
ontology:Latitude rdf:type owl:Class ;
                rdfs:subClassOf ontology:City .


###  http://example.com/ontology#Longitude
ontology:Longitude rdf:type owl:Class ;
                 rdfs:subClassOf ontology:City .


###  http://example.com/ontology#Low
ontology:Low rdf:type owl:Class ;
           rdfs:subClassOf ontology:ActivityStatus .


###  http://example.com/ontology#Max_Temp
ontology:Max_Temp rdf:type owl:Class ;
                rdfs:subClassOf ontology:ExternalWeatherData .


###  http://example.com/ontology#Medium
ontology:Medium rdf:type owl:Class ;
              rdfs:subClassOf ontology:ActivityStatus .


###  http://example.com/ontology#Min_Temp
ontology:Min_Temp rdf:type owl:Class ;
                rdfs:subClassOf ontology:ExternalWeatherData .


###  http://example.com/ontology#Mobile
ontology:Mobile rdf:type owl:Class ;
              rdfs:subClassOf ontology:PersonalInformation .


###  http://example.com/ontology#Motivational
ontology:Motivational rdf:type owl:Class ;
                    rdfs:subClassOf ontology:RecommendationGeneration .
```

```
###  http://example.com/ontology#Name
ontology:Name rdf:type owl:Class ;
          rdfs:subClassOf ontology:City .


###  http://example.com/ontology#Outdoor
ontology:Outdoor rdf:type owl:Class ;
             rdfs:subClassOf ontology:ActivityType .


###  http://example.com/ontology#PersonalInformation
ontology:PersonalInformation rdf:type owl:Class ;
                     rdfs:label "Personal Information" .


###  http://example.com/ontology#PersonalPreferences
ontology:PersonalPreferences rdf:type owl:Class ;
                     rdfs:label "Personal Preferences" .


###  http://example.com/ontology#Pressure
ontology:Pressure rdf:type owl:Class ;
             rdfs:subClassOf ontology:ExternalWeatherData .


###  http://example.com/ontology#Real_Feel
ontology:Real_Feel rdf:type owl:Class ;
              rdfs:subClassOf ontology:ExternalWeatherData .


###  http://example.com/ontology#RecommendationGeneration
ontology:RecommendationGeneration rdf:type owl:Class ;
                         rdfs:label "Recommendation Generation" .


###  http://example.com/ontology#Sea_Level
ontology:Sea_Level rdf:type owl:Class ;
              rdfs:subClassOf ontology:ExternalWeatherData .


###  http://example.com/ontology#Sedentary_Bouts
ontology:Sedentary_Bouts rdf:type owl:Class ;
                  rdfs:subClassOf ontology:GoalStatus .


###  http://example.com/ontology#Sedentary_Time
ontology:Sedentary_Time rdf:type owl:Class ;
                  rdfs:subClassOf ontology:ActivityStatus .


###  http://example.com/ontology#Sleep_Time
ontology:Sleep_Time rdf:type owl:Class ;
               rdfs:subClassOf ontology:ActivityStatus .
```

```
###  http://example.com/ontology#Status
ontology:Status rdf:type owl:Class ;
            rdfs:subClassOf ontology:ExternalWeatherData .


###  http://example.com/ontology#Temperature
ontology:Temperature rdf:type owl:Class ;
                rdfs:subClassOf ontology:ExternalWeatherData .


###  http://example.com/ontology#Time
ontology:Time rdf:type owl:Class ;
          rdfs:subClassOf ontology:Timestamp .


###  http://example.com/ontology#Timestamp
ontology:Timestamp rdf:type owl:Class ;
              rdfs:label "Timestamp" .


###  http://example.com/ontology#Visibility
ontology:Visibility rdf:type owl:Class ;
               rdfs:subClassOf ontology:ExternalWeatherData .


###  http://example.com/ontology#Wind_Degree
ontology:Wind_Degree rdf:type owl:Class ;
                rdfs:subClassOf ontology:ExternalWeatherData .


###  http://example.com/ontology#Wind_Gust
ontology:Wind_Gust rdf:type owl:Class ;
              rdfs:subClassOf ontology:ExternalWeatherData .


###  http://example.com/ontology#Wind_Speed
ontology:Wind_Speed rdf:type owl:Class ;
               rdfs:subClassOf ontology:ExternalWeatherData .


###  Generated by the OWL API (version 4.5.9.2019-02-01T07:24:44Z) https://github.com/owlcs/owlapi
```
