# Supplementary material for: Contextual recommendation modeling in eCoaching with machine learning, X-AI, and semantic ontology
Source: Front Digit Health. 2026 Jul 15;8:1811976. doi: 10.3389/fdgth.2026.1811976 (PMC13416675; doi:10.3389/fdgth.2026.1811976)
Supplement: Supplementary file 6 [file Datasheet6.pdf]

In [1]:

```python
import numpy as np
import pandas as pd
from sklearn.model_selection import train_test_split
from sklearn.tree import DecisionTreeClassifier
from sklearn.metrics import accuracy_score
```

In [2]:

```python
# Step 1: Initialize Dummy Data
def generate_dummy_weather_data(num_days):
    """Generate dummy bi-hourly weather data."""
    data = {
        'Temperature': np.random.uniform(10, 35, num_days * 12),
        'Humidity': np.random.uniform(30, 80, num_days * 12),
        'WindSpeed': np.random.uniform(0, 15, num_days * 12),
        'Rainfall': np.random.choice([0, 1], num_days * 12, p=[0.8, 0.2]),
        'ActivityClass': np.random.choice(['Low', 'Moderate', 'High'], num_days * 1
    }
    return pd.DataFrame(data)
```

In [3]:

```python
# Generate dummy individual activity data
def generate_dummy_activity_data(num_days):
    """Generate dummy daily activity data."""
    data = {
        'Day': np.arange(1, num_days + 1),
        'ActivityScore': np.random.uniform(50, 100, num_days),
        'GoalScore': np.random.uniform(70, 100, num_days),
    }
    return pd.DataFrame(data)
```

In [4]:

```python
# Generate recommendation messages
def generate_recommendation_messages():
    """Return a set of dummy recommendation messages."""
    return ["Take a walk", "Go for a jog", "Do indoor yoga", "Cycling", "Swimming"]
```

In [5]:

```python
# Dummy SPARQL query processor
def sparql_query_processor(contextual_data):
    """Simulate SPARQL query processing."""
    return np.random.choice(generate_recommendation_messages(), size=1)[0]
```

In [6]:

```python
# Algorithm Simulation
def simulate_algorithm(num_days=2000):
    """Simulate the contextual activity recommendation algorithm."""
    # Initialize data
    weather_data = generate_dummy_weather_data(num_days)
    activity_data = generate_dummy_activity_data(num_days)
    recommendation_messages = generate_recommendation_messages()

    days = 0
    dueco = num_days
    recommendations = []

    while days < dueco:
        # Step 1: Load and preprocess data
        daily_weather = weather_data.iloc[days * 12:(days + 1) * 12]
```

```
        X = daily_weather.drop(columns=['ActivityClass'])
        y = daily_weather['ActivityClass']

        x_train, x_test, y_train, y_test = train_test_split(X, y, test_size=0.1, ra

        # Step 2: Train Classifier
        clf = DecisionTreeClassifier()
        clf.fit(x_train, y_train)
        y_pred = clf.predict(x_test)
        accuracy = accuracy_score(y_test, y_pred)

        # Step 3: Contextual Recommendation
        delta_1 = activity_data.iloc[days]['ActivityScore']
        delta_2 = activity_data.iloc[days]['GoalScore']
        delta_3 = daily_weather['Temperature'].mean()
        delta_4 = daily_weather['Rainfall'].mean()
        delta_5 = delta_1
        delta_6 = delta_2 - delta_5

        contextual_data = {
            "delta_1": delta_1,
            "delta_2": delta_2,
            "delta_3": delta_3,
            "delta_4": delta_4,
            "delta_6": delta_6,
        }

        recommended_message = sparql_query_processor(contextual_data)

        # Update recommendation list
        recommendations.append({
            "Day": days + 1,
            "Recommendation": recommended_message,
            "Accuracy": accuracy
        })

        days += 1

    return pd.DataFrame(recommendations)
```

In [7]:
```
# Run Simulation
recommendation_results = simulate_algorithm(num_days=7)
```

In [8]:
```
# Display Results
print(recommendation_results)
```

```
   Day  Recommendation  Accuracy
0    1     Take a walk       0.0
1    2     Take a walk       0.5
2    3     Take a walk       0.0
3    4        Swimming       1.0
4    5        Swimming       0.5
5    6        Swimming       0.0
6    7  Do indoor yoga       0.0
```

In [9]:

```python
# Test Cases
def test_algorithm():
    test_cases = [
        {
            "input": 3,
            "expected_output": "A DataFrame with recommendations for 3 days"
        },
        {
            "input": 5,
            "expected_output": "A DataFrame with recommendations for 5 days"
        },
        {
            "input": 1,
            "expected_output": "A DataFrame with recommendations for 1 day"
        },
        {
            "input": 10,
            "expected_output": "A DataFrame with recommendations for 10 days"
        },
        {
            "input": 0,
            "expected_output": "An empty DataFrame or error due to invalid input"
        }
    ]

    for i, case in enumerate(test_cases):
        try:
            result = simulate_algorithm(case["input"])
            assert len(result) == case["input"], f"Test case {i + 1} failed"
            print(f"Test case {i + 1} passed")
        except Exception as e:
            print(f"Test case {i + 1} failed: {e}")

# Run Test Cases
test_algorithm()
```

```
Test case 1 passed
Test case 2 passed
Test case 3 passed
Test case 4 passed
Test case 5 passed
```

In [ ]:
