# Supplementary material for: Contextual recommendation modeling in eCoaching with machine learning, X-AI, and semantic ontology
Source: Front Digit Health. 2026 Jul 15;8:1811976. doi: 10.3389/fdgth.2026.1811976 (PMC13416675; doi:10.3389/fdgth.2026.1811976)
Supplement: Supplementary file 7 [file Datasheet7.pdf]

Representative SPARQL Queries

The following representative SPARQL queries were used to validate ontology structure, retrieve contextual weather concepts, identify activity-type classes, verify recommendation-generation relations, and support the test cases presented in Table S-3 (ref. Supplementary Material - 3).

1. Retrieve all recommendation instances
   ```
   SELECT ?r
   WHERE {
   ?r a ontology:RecommendationGeneration .
   }
   ```
2. Retrieve all weather data classes
   ```
   SELECT ?w
   WHERE {
   ?w rdfs:subClassOf ontology:ExternalWeatherData .
   }
   ```
3. Retrieve all activity type classes
   ```
   SELECT ?a
   WHERE {
   ?a rdfs:subClassOf ontology:ActivityType .
   }
   ```
4. Retrieve indoor activity class
   ```
   SELECT ?a
   WHERE {
   ?a rdfs:subClassOf ontology:ActivityType .
   FILTER(?a = ontology:Indoor)
   }
   ```
5. Retrieve outdoor activity class
   ```
   SELECT ?a
   WHERE {
   ?a rdfs:subClassOf ontology:ActivityType .
   FILTER(?a = ontology:Outdoor)
   }
   ```
6. Retrieve all activity status classes
   ```
   SELECT ?s
   WHERE {
   ?s rdfs:subClassOf ontology:ActivityStatus .
   }
   ```
7. Retrieve low activity status
   ```
   SELECT ?s
   WHERE {
   ?s rdfs:subClassOf ontology:ActivityStatus .
   FILTER(?s = ontology:Low)
   }
   ```

8. Retrieve medium activity status
```
SELECT ?s
WHERE {
?s rdfs:subClassOf ontology:ActivityStatus .
FILTER(?s = ontology:Medium)
}
```
9. Retrieve high activity status
```
SELECT ?s
WHERE {
?s rdfs:subClassOf ontology:ActivityStatus .
FILTER(?s = ontology:High)
}
```
10. Retrieve all goal status classes
```
SELECT ?g
WHERE {
?g rdfs:subClassOf ontology:GoalStatus .
}
```
11. Retrieve user preference classes
```
SELECT ?p
WHERE {
?p a owl:Class .
FILTER(?p = ontology:PersonalPreferences)
}
```
12. Retrieve personal information classes
```
SELECT ?p
WHERE {
?p rdfs:subClassOf ontology:PersonalInformation .
}
```
13. Retrieve city-related classes
```
SELECT ?c
WHERE {
?c rdfs:subClassOf ontology:City .
}
```
14. Retrieve timestamp-related classes
```
SELECT ?t
WHERE {
?t rdfs:subClassOf ontology:Timestamp .
}
```
15. Retrieve recommendations with weather data
```
SELECT ?r ?w
WHERE {
?r ontology:hasWeatherData ?w .
}
```

16. Retrieve recommendations with activity type
```
SELECT ?r ?a
WHERE {
?r ontology:hasActivityType ?a .
}
```
17. Retrieve recommendations with goal status
```
SELECT ?r ?g
WHERE {
?r ontology:hasGoalStatus ?g .
}
```
18. Retrieve recommendations with activity status
```
SELECT ?r ?s
WHERE {
?r ontology:hasActivityStatus ?s .
}
```
19. Retrieve recommendations by timestamp
```
SELECT ?r ?t
WHERE {
?r ontology:hasTimestamp ?t .
}
```
20. Retrieve recommendations linked to personal information
```
SELECT ?r ?p
WHERE {
?r ontology:hasPersonalInformation ?p .
}
```
21. Retrieve city and preference relation
```
SELECT ?person ?city ?pref
WHERE {
?person ontology:hasCity ?city .
?person ontology:hasPreferences ?pref .
}
```
22. Retrieve actionable recommendation classes
```
SELECT ?r
WHERE {
?r rdfs:subClassOf ontology:RecommendationGeneration .
FILTER(?r = ontology:Actionable)
}
```
23. Retrieve motivational recommendation classes
```
SELECT ?r
WHERE {
?r rdfs:subClassOf ontology:RecommendationGeneration .
FILTER(?r = ontology:Motivational)
}
```

24. Retrieve weather features relevant to rainy-day recommendation
```
SELECT ?w
WHERE {
?w rdfs:subClassOf ontology:ExternalWeatherData .
FILTER(?w IN (
ontology:Description,
ontology:Humidity,
ontology:Cloud_All,
ontology:Visibility))
}
```
25. Retrieve weather features relevant to windy-day recommendation
```
SELECT ?w
WHERE {
?w rdfs:subClassOf ontology:ExternalWeatherData .
FILTER(?w IN (
ontology:Wind_Speed,
ontology:Wind_Degree,
ontology:Wind_Gust))
}
```
26. Retrieve weather features relevant to hot-day recommendation
```
SELECT ?w
WHERE {
?w rdfs:subClassOf ontology:ExternalWeatherData .
FILTER(?w IN (
ontology:Temperature,
ontology:Max_Temp,
ontology:Real_Feel,
ontology:Humidity))
}
```
27. Retrieve weather features relevant to snowy-day recommendation
```
SELECT ?w
WHERE {
?w rdfs:subClassOf ontology:ExternalWeatherData .
FILTER(?w IN (
ontology:Description,
ontology:Temperature,
ontology:Min_Temp,
ontology:Cloud_All))
}
```
28. Retrieve safety-sensitive weather features
```
SELECT ?w
WHERE {
?w rdfs:subClassOf ontology:ExternalWeatherData .
FILTER(?w IN (
```

```
ontology:Visibility,
ontology:Wind_Speed,
ontology:Wind_Gust,
ontology:Description))
}
```

29. Retrieve complete recommendation context

```
SELECT ?r ?w ?a ?g ?s ?t
WHERE {
?r ontology:hasWeatherData ?w .
?r ontology:hasActivityType ?a .
?r ontology:hasGoalStatus ?g .
?r ontology:hasActivityStatus ?s .
?r ontology:hasTimestamp ?t .
}
```

30. Retrieve ontology object-property structure

```
SELECT ?property ?domain ?range
WHERE {
?property a owl:ObjectProperty .
OPTIONAL { ?property rdfs:domain ?domain . }
OPTIONAL { ?property rdfs:range ?range . }
}
```
